# Drug-induced chromatin accessibility changes associate with sensitivity to liver tumor promotion

**DOI:** 10.26508/lsa.201900461

**Published:** 2019-10-15

**Authors:** Antonio Vitobello, Juliane Perner, Johanna Beil, Jiang Zhu, Alberto Del Río-Espínola, Laurent Morawiec, Magdalena Westphal, Valérie Dubost, Marc Altorfer, Ulrike Naumann, Arne Mueller, Karen Kapur, Mark Borowsky, Colin Henderson, C Roland Wolf, Michael Schwarz, Jonathan Moggs, Rémi Terranova

**Affiliations:** 1 Novartis Institutes for BioMedical Research (NIBR), Basel, Switzerland; 2 NIBR, Cambridge, MA, USA; 3 Inserm, Unité Mixte de Recherche (UMR) 1231, Université de Bourgogne-Franche Comté, Dijon, France; 4 School of Medicine, Jacqui Wood Cancer Centre, Ninewells Hospital and Medical School, University of Dundee, Dundee, UK; 5 Department of Toxicology, University of Tübingen, Tübingen, Germany; 6 Innovative Medicines Initiative MARCAR Consortium (http://www.imi-marcar.eu/index.php)

## Abstract

Liver cancer susceptibility varies amongst humans and between experimental animal models because of multiple genetic and epigenetic factors. The molecular characterization of such susceptibilities has the potential to enhance cancer risk assessment of xenobiotic exposures and disease prevention strategies. Here, using DNase I hypersensitivity mapping coupled with transcriptomic profiling, we investigate perturbations in *cis*-acting gene regulatory elements associated with the early stages of phenobarbital (PB)-mediated liver tumor promotion in susceptible versus resistant mouse strains (B6C3F1 versus C57BL/6J). Integrated computational analyses of strain-selective changes in liver chromatin accessibility underlying PB response reveal differential epigenetic regulation of molecular pathways associated with PB-mediated tumor promotion, including Wnt/β-catenin signaling. Complementary transcription factor motif analyses reveal mouse strain–selective gene regulatory networks and a novel role for Stat, Smad, and Fox transcription factors in the early stages of PB-mediated tumor promotion. Mapping perturbations in *cis*-acting gene regulatory elements provides novel insights into the molecular basis for susceptibility to xenobiotic-induced rodent liver tumor promotion and has the potential to enhance mechanism-based cancer risk assessments of xenobiotic exposures.

## Abstract (precis)

This work explores quantitative chromatin accessibility, transcriptional and *cis*-acting gene regulatory variations underlying mouse strain–specific differences in drug-induced liver tumor promotion sensitivity.

## Introduction

Hepatocellular carcinoma (HCC) is the most common primary malignancy of the liver. Chronic liver disease due to hepatitis B virus or hepatitis C virus and alcohol accounts for the most HCC cases, but HCC has a multitude of etiological risk factors, including the baseline genetic and epigenetic makeup and multiple environmental cues such as aflatoxin and exposure to chemicals and pharmaceuticals (Gariboldi et al, 1993; Dragani, 2010; Ghouri et al, 2017). Hepatocarcinogenesis is an extremely complex, multistep process involving prominent environment-induced genetic and epigenetic alterations ultimately leading to malignant transformation of the hepatocytes (Farber, 1984; Pogribny & Rusyn, 2014). Although key pathways associated with HCC initiation are emerging, the molecular basis for strain-, gender- or species susceptibilities to HCC is still poorly understood. The identification of the underlying mechanism-based molecular alterations that drive hepatocyte transformation and promote development and progression of HCC is critical for its detection, therapeutic intervention, prevention, and for cancer risk assessment of chemical and pharmaceutical products.

Epigenetic variation, together with genetic variation and meta-genomic variation, represent key drivers of phenotypic variation in health and disease. Notably, epigenetic signatures are strongly influenced by the environment and determine phenotypic responses in health and disease (Jirtle & Skinner, 2007; Feil & Fraga, 2012). Epigenetic alterations can occur during the early stages of malignancy, plausibly remaining latent until further stimulation by endogenous or environmental factors (Feinberg et al, 2016; Vicente-Duenaset al, 2018). Multiple layers of epigenetic marks and mechanisms control genome function (Allis & Jenuwein, 2016). Methods for profiling chromatin accessibility, including DNase I hypersensitivity mapping, enable the identification of *cis*-acting gene regulatory elements (or cistrome) that are important for determining cell type identity and functions. The cistrome includes well-characterized DNA sequence elements such as enhancers and promoters and is strongly enriched for transcription factor (TF)-binding sites, providing critical genome regulatory information, but not limited to gene expression regulation (Consortium, 2012; Shen et al, 2012; Thurman et al, 2012; Johanson et al, 2018). Notably, recent integration of regulatory DNA and disease- and trait-associated genetic variant catalogs have demonstrated a disproportionate (>80%) enrichment of disease-associated genetic variants in noncoding enhancer regions. The genetic variants were reported to disrupt important cell type–specific TF regulatory interactions (Maurano et al, 2012; Hnisz et al, 2013; Pennacchio et al, 2013; Mifsud et al, 2015) and were associated with a broad range of phenotypic effects, sometimes driven by subtle effects on target gene expression (Corradin et al, 2014; Soldner et al, 2016). The perturbation of regulatory regions, including enhancers, plays an important role during the tumorigenic process (Davie et al, 2015; Sur & Taipale, 2016). Thus, mapping such perturbations during spontaneously occurring or environmentally driven carcinogenesis has high potential for identifying early mechanism-based biomarkers of carcinogenesis (Thomson et al, 2014; Luisier et al, 2014b).

Several rodent models have been used in defining the pathogenesis of HCC and have contributed to the current knowledge of HCC (Heindryckx et al, 2009; Santos et al, 2017). Treatment of mice with phenobarbital (PB) represents one of the best characterized models of xenobiotic-induced liver tumor promotion and has been extensively evaluated to investigate the kinetics and molecular drivers associated with drug-induced rodent non-genotoxic hepatocarcinogenesis. We previously reported that PB-mediated liver tumor promotion is accompanied by significant progressive transcriptional, epigenetic (DNA methylome and hydroxymethylome), and TF regulatory changes preceding liver-specific tumorigenic events, some of the changes plausibly reflecting dedifferentiation/reprogramming of hepatocytes towards a stem cell–like state (Bachman et al, 2006; Phillips et al, 2009a; Lempiainen et al, 2011, 2013; Thomson et al, 2012, 2016; Luisier et al, 2014b).

Although commonly considered a “rodent” non-genotoxic liver carcinogen, significant species and strain differences (qualitative and quantitative) have been observed for PB-mediated liver tumor promotion, highlighting preexisting differences in baseline tumor susceptibilities (Table S1) (Peraino et al, 1973; Becker, 1982; Diwan et al, 1986; Goldsworthy & Fransson-Steen, 2002). PB positively selects for β-catenin (*Ctnnb1*)-mutated liver tumors in the mouse, although inhibiting the outgrowth of mouse liver tumors that harbor an activated MAPK-pathway (i.e., *Ha-ras* or *B-raf*–mutated) (Aydinlik et al, 2001; Calvisi et al, 2004). Sensitive mouse strains (e.g., C3H or the hybrid strain B6C3F1) develop *Ctnnb1*-mutated neoplasms within 12 mo of PB exposure with high incidence, whereas tumor-resistant strains (e.g., C57BL/6) only develop liver tumors after an initiating mutagenic event and long-term PB exposure (Table S1 and references within).

Table S1 Incidence (number of affected animals per group [x/y] and % are indicated) of macroscopical liver tumors from three independent cross-strain comparison studies indicate that strain differences in hepatocarcinogenesis sensitivity exist among mice with different genetic backgrounds. C3H and B6C3F1 reproducibly show higher sensitivity to liver tumor promotion than C57BL/6. Time course study data from Bursch et al. also indicate that although C57BL/6 may develop tumors after diethylnitrosamine (DEN) initiation, the effects are delayed compared with other strains. Whereas the three studies consistently point to cross-strain differences in liver tumor promotion, several experimental variables may contribute to differences in phenotypic outcome and should be noted. 1) Sub-strain differences across studies (use of C57BL/6N [MGI:2159965] or C57BL/6J [MGI:3028467]; use of C3H/HeN [MGI:2160972] or C3H/He [MGI:2159866]); presumably extending to the hybrid strains B6C3F1. 2) Treatment conditions: PB treatment was done in comparable conditions, with minimal variation in dose or route. However, major differences in DEN initiation are reported by Goldsworthy & Fransson-Steen (DEN delivered IP at 1 mg/kg) and Bursch et al. (DEN delivered IP at 90 mg/kg). 3) Age of animals at onset of treatment was also significantly different across studies (Study by Becker: 6–8-wk-old animals, study by Goldsworthy & Fransson-Steen: 15-d-old animals, study by Bursch et al.: 5-wk-old mice) and may constitute important variable in these studies. n.d., no data available; n.a., no animal at this time point.

At the molecular level, divergent transcriptional signatures have been identified in mouse strains exhibiting differential sensitivity to PB-driven tumor promotion effects (Phillips et al, 2009b), further supporting the impact of genetic and epigenetic variation on TFs and gene regulatory networks responsible for differential transcriptional readouts in response to PB.

Here, we have exploited established mouse strain–specific differences in sensitivity to PB-mediated tumor promotion to explore drug-induced chromatin regulatory and transcriptional variations underlying phenotypic responses at early stages of liver tumor promotion. We used genome-wide DNase I hypersensitivity profiling (DNase-seq) of liver tissue after treatment of mice with tumor-promoting doses of PB in both tumor-resistant C57BL/6J and tumor-prone hybrid B6C3F1 (C57BL/6 female × C3H/He male) strains. Our analysis reveals quantitative strain differences in hepatic molecular responses to PB at both transcriptional and chromatin accessibility levels, including genes that regulate Wnt/β-catenin signaling. Most PB-mediated chromatin accessibility changes occurred at distal intergenic (IG) regions that were on average ∼45 kb away from the nearest gene transcriptional start site (TSS), plausibly mapping to the location of *cis*-acting gene regulatory elements. The analysis of TF motifs underlying these predominantly strain-selective changes in chromatin accessibility highlights several novel candidate transcriptional co-regulators that may underlie the sensitivity of B6C3F1 mice to PB-mediated liver tumor promotion. These data also highlight the significant potential of mapping tissue-specific changes in *cis*-acting gene regulatory elements for providing novel insights into the molecular basis of xenobiotic-induced phenotypes, including the potential to enhance mechanism-based cancer risk assessments of xenobiotic exposures.

## Results

### Experimental model for comparing mouse strain sensitivity to PB-mediated tumor promotion

**Figure S1.**
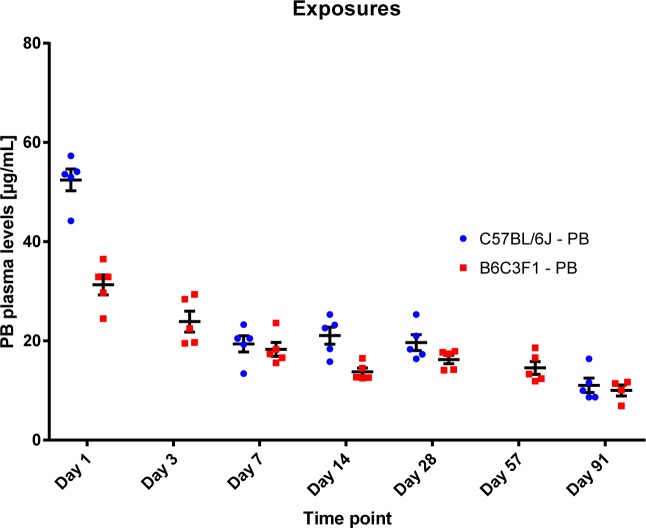
Kinetics of PB plasma concentrations over the full 91-d treatment period (assessed at day 1, 3, 7, 14, 28, 57, and 91) show equivalent PB exposure over time. Exposure levels (in μg/ml) were determined by liquid chromatography–mass spectrometry.

Molecular profiling of well-characterized mouse strains that exhibit differences in sensitivity to PB-mediated liver tumor promotion represents an ideal model system for identifying early mechanism-based markers of drug-induced liver tumorigenesis. We previously reported kinetic investigations of early hepatic pathological and transcriptional effects associated with PB treatment (ad libitum access to 0.05% [wt/vol] in drinking water for up to 91 d of treatment) in B6C3F1 (Lempiainen et al, 2013) and C57BL/6J (Luisier et al, 2014a) mouse strains. In these studies, PB concentrations in plasma and liver were determined by liquid chromatography–mass spectrometry and showed comparable and stable plasma levels of PB over time (Fig S1). This treatment regimen reportedly promotes high incidence of tumor formation exclusively in the B6C3F1 mouse strain in absence of mutagenic priming events (Table S1). Despite such differences in tumor promotion effects after long-term (≥1 yr) treatment, the liver histopathology phenotype induced by treatment at earlier time points was essentially identical in both strains and limited to hepatocellular hypertrophy (primarily of perivenous hepatocytes in the central zone of the lobule), starting from 8 d of PB treatment and increasing in severity at later time points (Lempiainen et al, 2013; Luisier et al, 2014a). Thus, this experimental setup enables the investigation of chromatin and transcriptional effects underlying early events of liver tumor promotion sensitivity.

### Differential chromatin accessibility maps of PB-treated mouse livers

**Figure 1.**
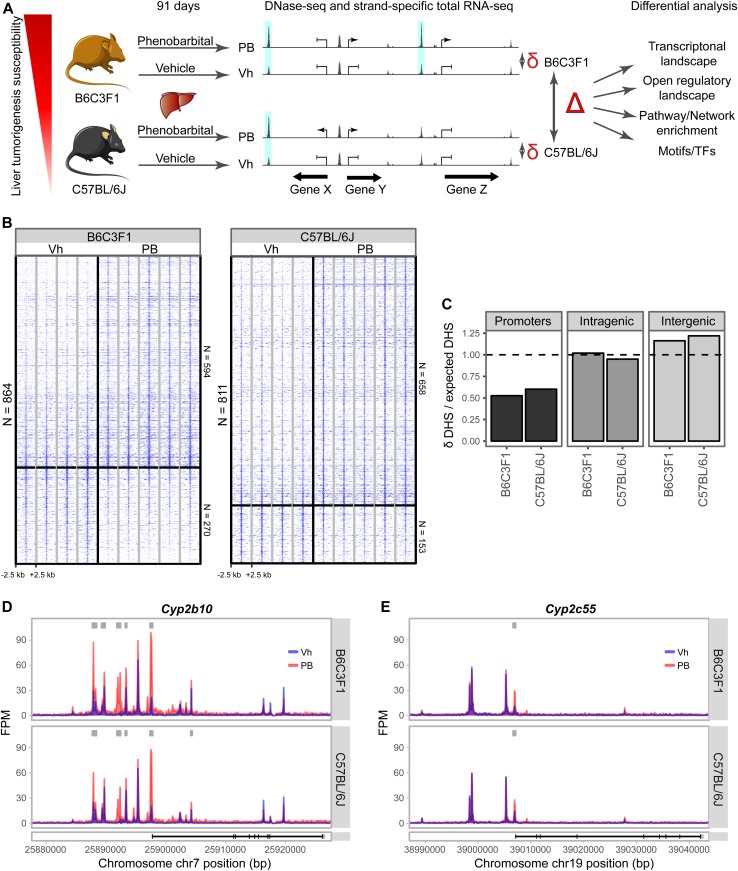
DNase hypersensitivity mapping of PB-treated mouse (B6C3F1 and C57BL/6J) livers. **(A)** Overview of experimental design and analysis plans. Mouse strains of differential tumor promotion sensitivity were PB-treated for 91 d in comparable conditions and with comparable histopathological outcomes. Their livers were processed to profile transcriptome (RNA-seq) and open chromatin landscape (DNase-seq). PB response effects (δ) are compared across strains (Δ) using different computational approaches. **(B)** Profiles of PB-mediated open chromatin changes (|log2 FC| ≥ 0.58, FDR < 0.01) in livers of B6C3F1 and C57BL/6J. Windows of 5 kb (−2.5 to +2.5 kb) centered around each identified δ-DHS are illustrated. Log2-transformed DHS fragment counts in consecutive 50-bp bins within the 5-kb window scaled to average library size are shown. Color intensity from low to high represents signals within the range (1.5, 7). Individual samples are separated by grey lines. **(C)** Relative genomic distribution of δ-DHS with respect to UCSC genome annotation. **(D, E)** Genome browser tracks show δ-DHS effects at the promoter and proximal regulatory regions of two PB-responsive genes *Cyp2b10* and *Cyp2c55*.

We hypothesized that the mapping of PB treatment-mediated hepatic transcriptional and epigenetic effects (δ = PB response), and their comparison across mouse strains (Δ = strain-selective PB effects) may enable the characterization of key gene regulatory elements and associated TFs underlying differential tumorigenicity outcomes after chronic PB exposure (Fig 1A).

Mapping chromatin accessibility landscapes can help elucidate key genome regulatory regions (e.g., gene promoters and enhancers) and associated epigenetic mechanisms of gene regulation (Consortium, 2012; Thurman et al, 2012). We, thus, profiled gene expression and chromatin accessibility landscapes in tumor-resistant C57BL/6J and hybrid tumor-prone B6C3F1 male mice after 91 d of PB treatment using archived tissues from our previously reported kinetic studies (Lempiainen et al, 2013; Luisier et al, 2014a).

**Figure S2.**
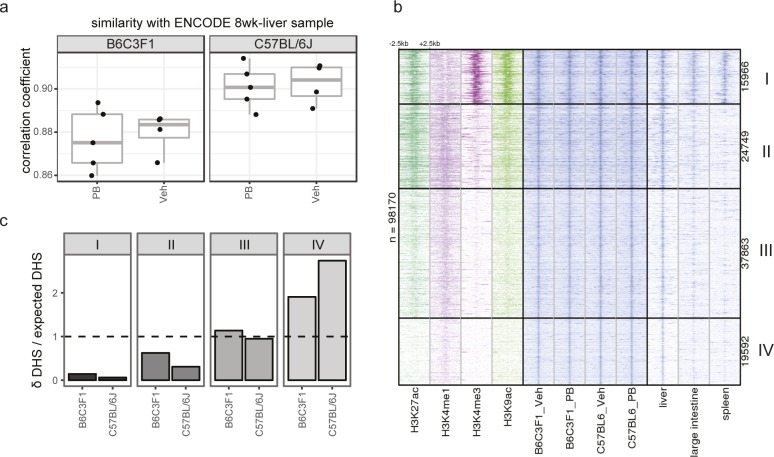
Quality control statistics for DNase hypersensitivity mapping of PB-treated mouse (B6C3F1 and C57BL/6J) livers. **(A)** Correlation coefficients of our liver DHS profiling data with previously reported, publicly available liver ENCODE data, highlighting high levels of correlation. **(B)** Genome-wide DHS data are compared with liver ENCODE data and with non-relevant tissues (large intestine and spleen), illustrating the overall lineage consistence of the open chromatin landscape. Liver chromatin marks H3K27ac, H3K4me1, H3K4me3, and H3K9ac were also aligned, highlighting different chromatin behavior clusters and associated functional genomics landscapes (cluster I: constitutively open active promoter regions, cluster II: active enhancers, and cluster III and IV: poised/inactive enhancers or other regulatory regions). DHS regions were k-means clustered based on total log2-transformed CPM signal of each histone modification within the 1-kbp region centered at the DHS peak midpoint. **(C)** Associating δ-DHS to highlighted functional genomic regions shows enrichment for poised and inactive enhancer/or other regulatory regions (clusters III and IV). Some strain-selective differences are plausible at clusters II and IV.

**Figure S3.**
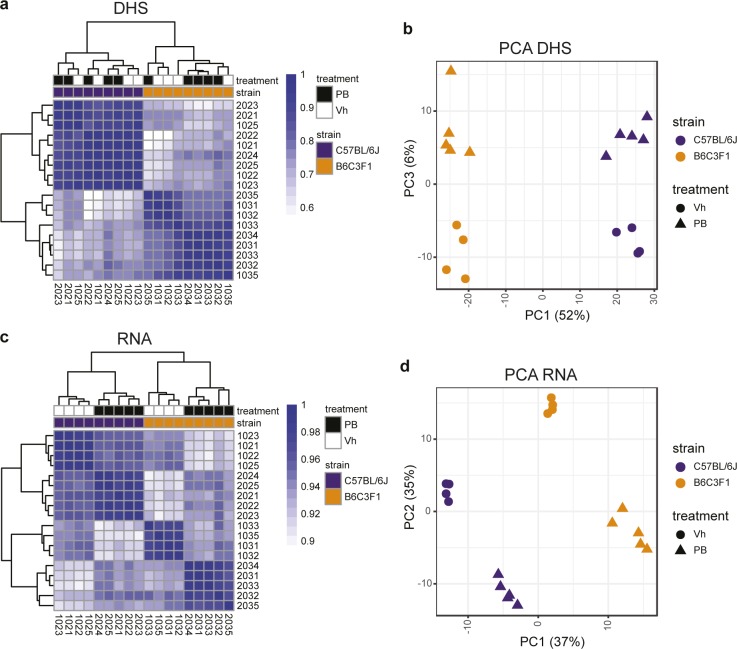
Correlation and principle component analysis (PCA) of DNase hypersensitivity mapping and RNAseq of PB treated mouse (B6C3F1 and C57BL/6J) livers. **(A)** Correlation matrix of DHS signals from individual samples, showing overall high reproducibility across samples with Pearson’s correlation coefficients above 0.55 for any pair-wise comparison. The dendrograms show the outcome of hierarchical clustering performed on the correlation matrix using Euclidian distance and complete linkage. Baseline strain-selective effects and variable overall PB treatment effects become apparent. **(B)** PCA of DHS signals showing separation by strain in PC1 and by treatment in PC3. **(C)** Correlation matrix of RNA signals from individual samples, showing overall high reproducibility across samples with correlation coefficients above 0.9 for any pair-wise comparison. Samples are clustered by strain and PB treatment. **(D)** PCA of RNA signals showing a separation by PB treatment and strain.

To gain insight into the gene regulatory landscape accounting for PB-mediated responses, we performed DNase I digestion combined with high-throughput sequencing (DNase-seq) on individual liver samples isolated from control and treatment groups (n = 4–5) of C57BL/6J and B6C3F1 mice. A consensus set of 98,170 DNase I hypersensitive sites (DHSs) was identified that show significant DHS signal in at least three samples. The obtained DNase-seq profiles are overall comparable with 8-wk-old mouse liver DNase-seq publicly available from ENCODE (Pearson’s correlation coefficients 0.86–0.91; Fig S2A). DHS signals from individual samples show high degree of reproducibility across samples (Pearson’s correlation coefficients 0.55–0.98). The unsupervised clustering and Principal Component Analysis (PCA) based on the top 5,000 most variable DHSs (Fig S3A and B) show that strain background (PC1) is the strongest source of variation among samples, whereas the treatment effects is less strong but consistent (PC3). Overall, this indicates that DNase-seq is a robust method to detect strain- and PB-mediated variations in accessible chromatin regions genome wide.

Using library size–adjusted read counts at each DHS as direct quantitative readout of chromatin accessibility, we performed differential DHS analysis between vehicle- and PB-treated sample groups (δ-DHS). Using |log2 Fold Change (FC)| ≥ 0.58 and False Discovery Rate (FDR) ≤ 0.01 as cutoffs, we found a comparable number of PB-mediated differentially accessible δ-DHSs in B6C3F1 (n = 864) and C57BL/6J (n = 811) and comparable direction of increased/reduced chromatin accessibility effects across strains (Fig 1B and Table S2). Interestingly, we identified δ-DHSs at both promoter and upstream regulatory regions of well-characterized transcriptionally induced PB-responsive and constitutive androstane receptor (CAR)–regulated target genes, such as *Cyp2b10* and *Cyp2c55* (Honkakoski & Negishi, 1997; Konno et al, 2010; Lempiainen et al, 2011) (Fig 1D and E).

Table S2 DNase hypersensitivity sites, their statistics in the differential analysis, and the association to the nearest TSS for DHS peak sets identified as differential between vehicle and PB treatment. DHSs_all: Genomic position of all 98,170 consensus DHS regions with statistics for the different comparisons in the differential analysis. shared: Differential DHS peaks overlapping between B6C3F1 and C57BL/6J and their assignment to the nearest TSS. The table includes information on the peak location, the nearest gene loci definition, and the closest TSS. B6C3F1_enriched: Differential DHS peaks uniquely identified in B6C3F1 and their assignment to the nearest TSS. The table includes information on the peak location, the nearest gene loci definition, and the closest TSS. C57BL6J_enriched: Differential DHS peaks uniquely identified in C57BL/6J and their assignment to the nearest TSS. The table includes information on the peak location, the nearest gene loci definition, and the closest TSS.

To investigate which fraction of the functional genome was most affected by treatment-related changes, we first mapped the δ-DHSs to annotated promoters, intragenic and intergenic regions. We found overall enrichment of the δ-DHSs in intergenic regions and under-representation at promoter regions (here defined as the 1,000-bp region upstream of the Transcriptional Start Site (TSS) (Fig 1C), indicating that chromatin accessibility changed mostly at intergenic regulatory elements. Next, we investigated the activity status of the δ-DHSs at baseline using the histone modification profiles (H3K4me3, H3K4me1, H3K27ac, and H3K9ac) from the 8-wk-old mouse liver in ENCODE, which showed chromatin accessibility profiles consistent with our data. The integration of these four histone modification readouts to the open DHS landscape enables a functional partitioning of the genomic landscape (including constitutively opened promoter regions and tissue-specific active, poised, or silent enhancer regions) (Ram et al, 2011; Shlyueva et al, 2014). Aligning the histone modification profiles to the consensus set of DHSs, thus, revealed expected functional clustering of promoters (cluster I), active (cluster II), poised (cluster III), and inactive enhancers (cluster IV) (Fig S2B). This analysis revealed that the PB-mediated changes in open chromatin landscape in both strains were strongly enriched in cluster IV, whereas depleted at active chromatin clusters I and II (Fig S2C). We, however, noted that functionally active enhancer regions (cluster II) tended to show stronger enrichment for B6C3F1 over C57BL/6J, suggestive of a strain-specific effect in PB-mediated δ-DHS distribution.

Overall, our DNase-seq data robustly identifies a landscape of PB treatment–related regulatory chromatin changes. In both mouse strains, most δ-DHSs indicate increased chromatin accessibility and are enriched in intergenic regions, plausibly representing poised or inactive enhancer regions in the mouse liver.

### Quantitative strain differences in PB-mediated liver chromatin accessibility changes

**Figure 2.**
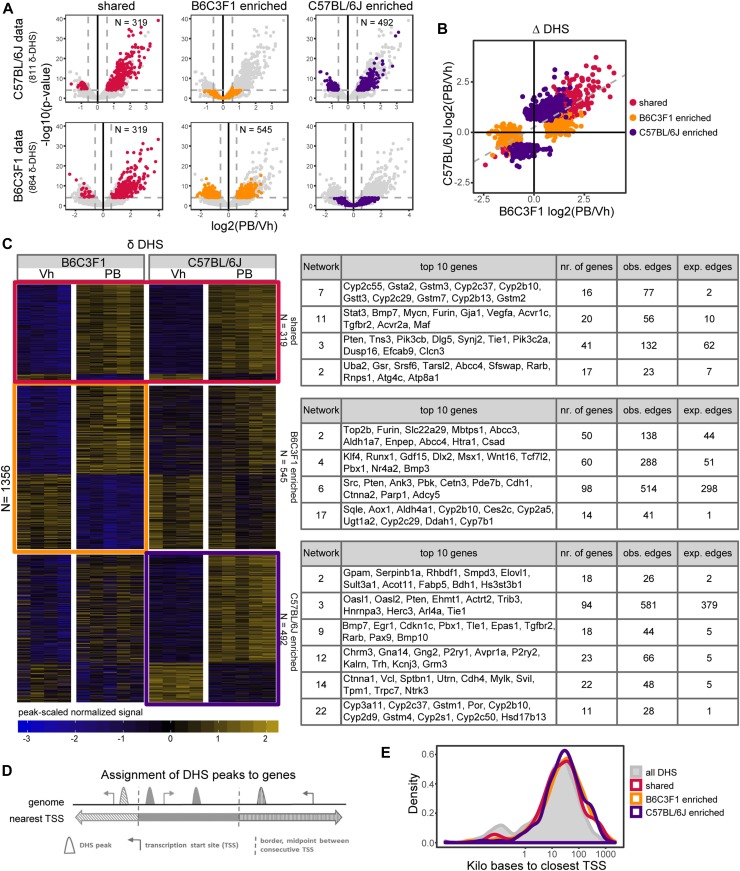
Quantitative strain-selective chromatin accessibility effects (Δ-DHS) upon PB treatment in mice. **(A)** Volcano plot showing the log2 FC and *P*-values of all genes (light grey) from the differential tests using the C57BL/6J samples (first row) or the B6C3F1 samples (second row). The 319 common (red), 545 B6C3F1-enriched (orange) and 492 C57BL/6J-enriched (purple) differentially expressed genes are highlighted. Dashed lines indicate selected statistical cutoffs (|log2 FC| = 0.58 and *P*-value corresponding to FDR = 0.01). **(B)** Linear correlation of Log2 FC PB treatment DHS effects across strains. Only Δ-DHS effects supported by either strain are shown and used to fit the linear regression line indicated by the dashed line. **(C)** Head-to-head comparison of PB-mediated δ-DHS effects, highlighting shared (n = 319, red box) and unique strain effects (n = 545 for B6C3F1, orange box and n = 492 for C57BL/6J, purple box). Δ-DHS effects were mapped to the nearest genes and STRING-db protein–protein interaction sub-networks enriched in each group are indicated (full list available from Table S3). **(D)** Illustrative summary of DHS peaks to gene assignment approach. A single gene was associated to each Δ-DHS through associating the nearest TSS. The full list of DHS-associated genes is available from Table S2. **(E)** Density distribution of distances to nearest TSS for genome-wide DHS landscape compared with PB-mediated δ−DHS for each indicated categories.

To evaluate the consistency of global chromatin accessibility effects in both strains, we projected the δ-DHSs (from Fig 1B) over the consensus DHS landscape in both strains (Fig 2A). We found that a subset of δ-DHSs is common to both strains (n = 319). These “shared” effects tend to occur in regions of strongest DHS log2 FC (Fig 2B) and are most consistent across replicates (Fig 2C). A comparable number of δ-DHSs only reached the cutoffs in one of the strains, with 545 and 492 δ-DHSs selectively enriched in B6C3F1 and C57BL/6J, respectively (Fig 2A). Direct visual comparison of the DHS landscapes (Fig 2C) points to coherent trends of the selectively enriched chromatin accessibility changes in both strains. Still, the differential analysis highlights differences of the effects in magnitude and/or consistency across replicates (only reaching the statistical thresholds in one of the strains). Taken together, the observed δ-DHSs are consistent with discrete cell-autonomous differences in molecular regulation and/or changes in small subsets of liver cells, rather than large changes in tissue composition or lineage identity after PB treatment.

Many coding genes are well annotated with their biological functions. Noncoding regions, however, typically lack such annotation. We next associated each Δ-DHS to the nearest TSS (Fig 2D), which is on average 45 kb away and can be located over 1,000 kb away (Fig 2E). Notably, whereas the shared DHSs follow the same bimodal (TSS and distal) distribution as the whole-genome DHS landscape, the strain-selective Δ-DHS landscape tends to distribute away from TSS, possibly accounting for distal enhancer-based changes (Fig 2E). This DHS–TSS proximity calling enabled us to build gene lists for each shared or strain-selective DHS group among which several well-known gene targets of PB signaling were identified (Table S2).

Acknowledging the limitations of DHS–TSS proximity calling and the risk that a fraction of gene hits may not be biologically relevant, we further analyzed the gene hits to identify common underlying biological functions within each shared or strain-selective gene group. We performed network enrichment analysis using the Search Tool for the Retrieval of Interacting Genes/Proteins (STRING database) (Jensen et al, 2009) to identify enrichment in protein–protein interaction partners. Consistent with the well-characterized PB-mediated hepatic xenobiotic response, STRING-db sub-networks enriched in shared DHS–TSS highlighted phase I metabolism genes (e.g., *Cyp2b10*, *Cyp2c55*, etc.) as well as signal transduction genes (e.g., *Stat3*, *Pten*, etc.) (Fig 2A and Table S3). The most connected genes in the STRING-db sub-networks enriched in B6C3F1 Δ-DHS–associated genes pointed out important regulatory factors, including *Klf4*, a key regulator of stem cell pluripotency (Takahashi et al, 2007; McConnell & Yang, 2010), Wnt/β-catenin signaling genes such as *Wnt16* and *Tcf7l2* (alias *Tcf4*) (network 4), and *Src *(network 6), which was previously reported to contribute PB-mediated mode-of-action, including through β-catenin signaling regulation (Groll et al, 2016). The C57BL/6J Δ-DHS group showed unique *Ctnna1* (network 14) enrichment and other effects related to metabolism, cell cycle, and differentiation (Fig 2A and Table S3).

**Figure 3.**
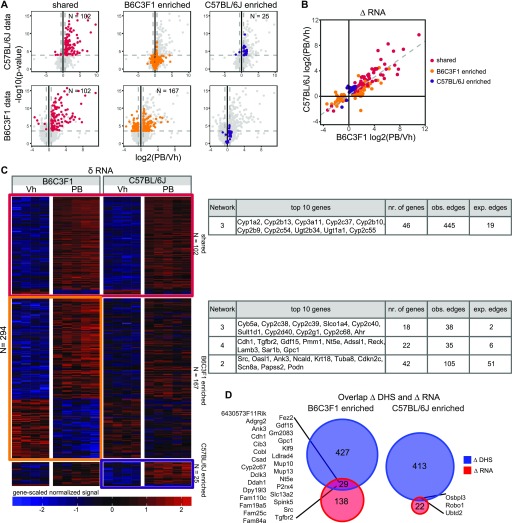
Strain-selective transcriptional effects (Δ-RNA) upon PB treatment in mice. **(A)** Volcano plot showing the log2 FC and *P*-values of all genes (light grey) from the differential tests using the C57BL/6J samples (first row) or the B6C3F1 samples (second row). The 102 common (red), 167 B6C3F1-enriched (orange), and 25 C57BL/6J-enriched (purple) differentially expressed genes are highlighted. Dashed lines indicate selected statistical cutoffs (|log2 FC| = 0.58 and *P*-value corresponding to FDR = 0.01). **(B)** Linear correlation of Log2 FC PB treatment RNA effects across strains. Only Δ-RNA effects supported by either strain are shown and used to fit the linear regression line indicated by the dashed line. **(C)** Head-to-head comparison of PB-mediated δ-RNA effects, highlighting shared (n = 102, red box) and unique strain effects (n = 167 for B6C3F1, orange box and n = 25 for C57BL/6J, purple box). The list of transcriptionally modulated genes is available from Table S4, STRING-db protein–protein interaction sub-networks enriched in each group are indicated (full list available from Table S3). **(D)** The overlay of Δ-DHS–associated genes (nearest TSS assignment approach) and Δ-RNA expression changes highlights a short list of loci displaying both changes in chromatin accessibility and transcriptional modulation, which are unique to each strain.

Table S3 Results of the STRING-db protein–protein interaction sub-network enrichment analyses using gene lists from DHS or RNA differential analysis. The table lists the number of genes in the identified sub-networks, the number of observed protein–protein interactions in the STRING-db between these genes, the number of expected interactions given a gene set of this size, the associated *P*-value and the list of genes ordered by their node degree. Only sub-networks supported by at least one direct interaction between genes are shown. Networks are ordered by *P*-value. Fig2_DHS_shared_networks: Networks identified using genes in shared DHS peaks of B6C3F1 and C57BL/6J. Fig2_DHS_B6C3F1_enriched_net: Network results using genes identified by TSS proximity of DHS peaks uniquely found in B6C3F1. Fig2_DHS_C57BL6J_enriched_net: Network results using gene identified by TSS proximity of DHS peaks uniquely found in C57BL/6J. Fig3_RNA_shared_networks: Networks identified using genes showing differential expression in both strains. Fig3_RNA_B6C3F1_enriched_net: Networks identified using genes showing differential expression uniquely in B6C3F1. Fig3_RNA_C57BL6J_enriched_net: Networks identified using genes showing differential expression uniquely in C57BL/6J.

**Figure S4.**
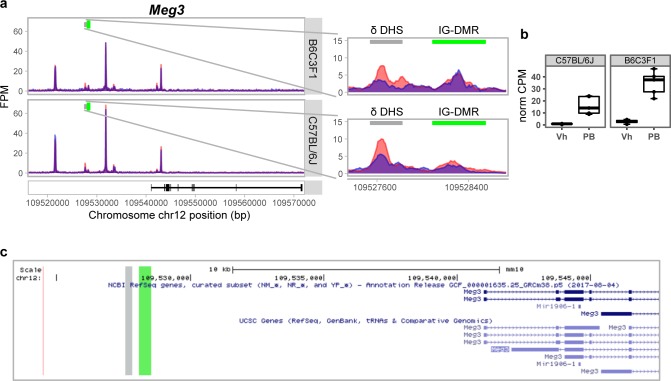
Strain-specific delta-DHS at *Meg3* within the *Dlk1-Dio3* imprinted locus **(A)** Genome browser DHS tracks show B6C3F1 predominant Δ-DHS effect at the *Dlk1-Dio3* cluster noncoding RNA gene *Meg3* locus (B6C3F1: log2 FC = 0.97, FDR = 0.0046; C57BL/6J: log2 FC = 0.48, FDR = 0.22). The identified region is in proximity but not overlapping to IG-DMR as defined in (51). **(B)** *Meg3* RNA-seq expression in CPM (B6C3F1 log2 FC = 3.6, FDR = 4.91E-6; C57BL/6J log2 FC = 4.3, FDR = 1.25E-5). **(C)** UCSC browser snapshot illustrating the genomic location of the DHS (grey) and *Dlk1-Dio3* cluster IG-DMR (green).

We previously reported CAR and Wnt/β-catenin signaling–dependent transcriptional effects at the pluripotency-associated *Dlk1-Dio3* imprinted gene cluster noncoding RNAs in the liver of mice treated with tumor-promoting doses of PB (Lempiainen et al, 2013; Pouche et al, 2017). Importantly we identify a minimal, but significant B6C3F1-specific increase in chromatin accessibility within the *Dlk1-Dio3* cluster upstream of the *Meg3* locus and at close proximity (within 500 bp) to the reported imprinting control region (Nowak et al, 2011), consistently we also measured enhanced *Meg3* transcriptional up-regulation in B6C3F1 liver material (Table S2 and Fig S4).

### Strain-selective PB-mediated liver transcriptional changes

To enable further functionalization of the DHS open regulatory landscape effects, we ran genome-wide RNA sequencing from matching liver samples. The RNA sequencing data were strongly clustered per strain and treatment effects, and PCA analyses of the top 1,000 most variable genes also show that strain background (PC1) and PB response (PC2) together account for most of the variation in the data (Fig S3C and D). Using cutoffs of log2 FC ≥ 0.58 and FDR < 0.01, we identified 127 differentially expressed genes in C57BL/6J and 269 in B6C3F1 samples (Table S4). Akin to the DHS landscape, we find predominant transcriptional perturbrations in a strain-selective manner. 102 genes are commonly regulated and tend to affect genes associated with the strongest transcriptional expression changes, whereas 167 and 25 genes are selectively modulated in B6C3F1 and C57BL/6J, respectively (Fig 3A and B). The effects are qualitatively consistent across strain and replicates (Fig 3C), thus again highlighting that strain differences pertain to quantitative effects in these total liver analyses.

Table S4 Results of the differential RNA-seq analysis. For each comparison, the estimated fold-change, the *P*-value and the FDR are given by the gene. The first six columns list for each strain the results of the vehicle and PB-treated comparison. The final six columns list for either vehicle or PB-treated groups in the between-strain comparison.

**Figure 4.**
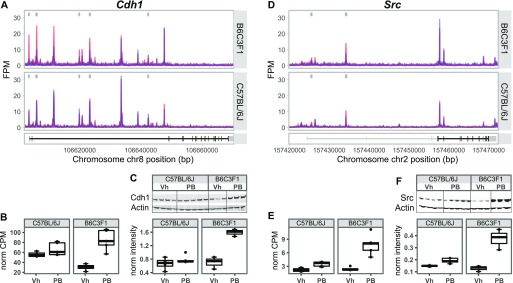
Strain-selective chromatin and transcriptional effects at Wnt/*β*-catenin signaling relevant to loci. **(A)** Genome browser DHS tracks show B6C3F1-enriched δ-DHS effects at the Cadherin-1 (*Cdh1*) locus. **(B)** RNA-seq, counts per million (CPM) data show B6C3F1-specific increase in *Cdh1* mRNA expression. **(C)** Western blot analysis and quantification show increase in Cdh1 protein expression in liver of PB-treated B6C3F1 animals. **(D)** Genome browser DHS tracks show B6C3F1-enriched Δ-DHS effects at the tyrosine-protein kinase *Src* locus. **(E)** RNA-seq, CPM data show B6C3F1-specific increase in *Src* mRNA expression. **(F)** Western blot analysis and quantification show increase in Src protein expression in the liver of PB-treated B6C3F1 animals. Each light grey box in the genomic browser snapshots represent a δ-DHS peak above statistical cutoffs (Log2 FC ≥ 0.58, FDR < 0.01).

Gene ontology (GO) term over-representation analyses showed enrichment for genes relevant to xenobiotic response in both strains (data not shown), whereas STRING-db protein–protein interaction enrichment analyses highlighted biologically significant B6C3F1-specific Δ-RNA changes. For example, of genes relevant to PB-mediated regulation of Wnt/β-catenin signaling (*Cdh1* or *Src* in networks 4 and 2) and other genes involved in important signaling functions in the liver such as *Tgfbr2* (network 4), a member of the canonical Smad-dependent TGF-β signaling cascade, involved in hepatic progenitor cell activation (Ding et al, 2013). As evidenced above by Δ-DHS/associated genes and RNA effects, some notable B6C3F1-specific effects are concordant, including *Src*, *Cdh1*, or *Tgfbr2*, and may represent predominantly strain-enriched signatures of functional relevance (compare network lists in Figs 2C and Figs 3C and see gene loci DHS and RNA effects illustration in Fig 4A and B [*Cdh1*] and Fig 4D and E [*Src*]).

### Strain-selective chromatin, transcriptional, and protein expression changes in the β-catenin pathway

**Figure S5.**
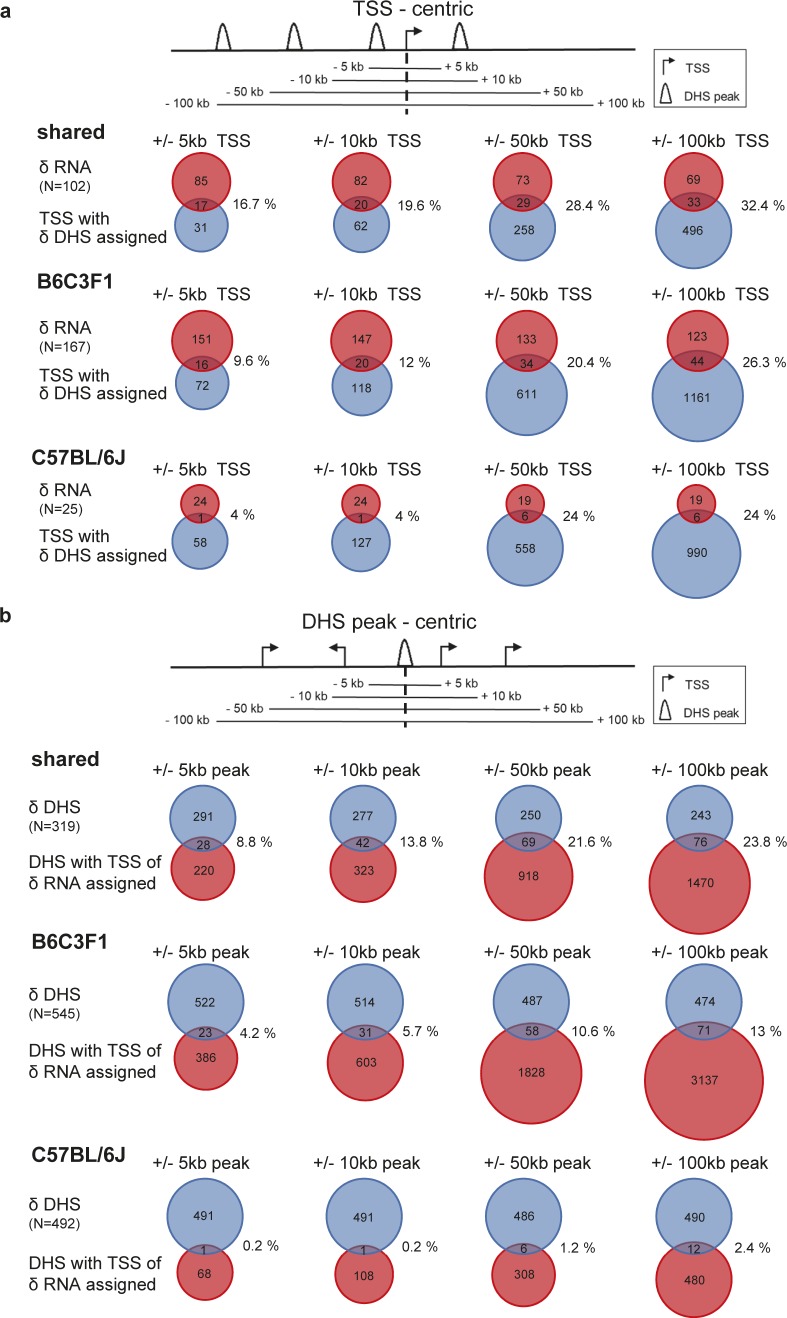
Complementary TSS-centric and DHS peak-centric approaches for assigning DHS peaks to gene regulation effects. **(A)** TSS-centric approach: each δ−DHS was assigned to regions of 10 to 200 kbp centered at the TSS (multiple assignments possible, see for example Fig 1D: *Cyp2b10* locus). The Venn diagrams were then generated from the TSS regions that have a δ-DHS assigned and the δ-RNA in the shared and strain-selective landscapes. **(B)** DHS-centric approach: The regions of 10 to 200 kbp were centered at DHS peaks and assigned with δ−RNA if their TSS falls into the region (multiple assignments possible). Venn diagrams were then generated from the DHS peaks that have a δ-RNA assigned and the δ-DHS.

**Figure 5.**
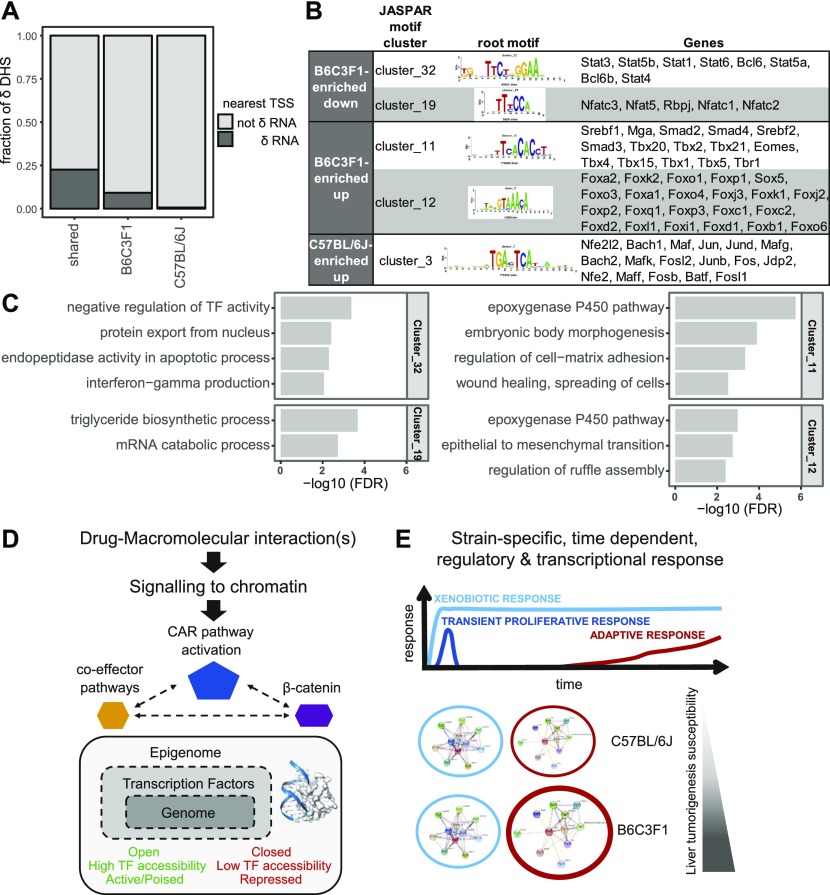
Strain-selective TF regulatory pathways effects upon PB treatment. **(A)** Most Δ-DHSs are distal to gene TSS and not associated with significant changes in the nearest gene expression (δ-RNA). **(B)** Summary of TF motif enrichment in down-regulated and up-regulated Δ-DHSs relative to the full consensus set of detected DHSs. Four clusters of motifs are shown for B6C3F1 and one for C57BL/6J (the full list available from Table S5). The genes shown are expression-filtered members of the motif cluster sorted by average expression. **(C)** GO biological pathway analysis of predicted TF target sites in the respective Δ-DHS group. **(D)** Updated model from Lempiainen et al (2013), Pouche et al (2017) highlighting the importance of the signaling to chromatin and novel potential TF regulatory pathways (baseline and treatment related) in response to CAR-activating xenobiotic exposure. **(E)** Predominantly strain-selective β-catenin and additional co-effector regulatory pathway effects (red) are likely contributing to the chronic and long-lasting adaptive response associated to increased tumor promotion sensitivity in B6C3F1 mice.

By systematically overlapping Δ-RNA and genes with nearby Δ-DHS changes, we characterized 29 of 167 (17% overlap, B6C3F1) and 3 of 25 (12% overlap, C57BL/6J) genes of concurrent RNA and DHS effects (Fig 3D). Because the chromatin at gene promoters is by and large constitutively opened and from the overall distal distribution of δ-DHSs (Figs 1 and S2B and C), a limited functional overlap between DHS/gene expression changes is expected. We also evaluated whether alternative DHS gene assignment approaches may yield different functional overlap output. Scanning the whole-genome TSS (n = 34,219) for δ-DHS association in flanking windows of ±5–100 kbp identified 10–26% functional differential RNA/DHS overlap in B6C3F1 and 4–24% overlap in C57BL/6J (Fig S5A). In a complementary approach, scanning the differential DHS landscape for δ-RNA association in flanking windows of ±5–100 kbp identified 4–13% functional RNA/DHS overlap in B6C3F1 and close to null in C57BL/6J (Fig S5B). The limited overlap in the later method is expected from the larger number of DHSs over gene expression changes in both strains. Thus, overall using a range of gene/DHS assignment approaches yields equivalent overlap, in the range of 10–20%. Interestingly, a large number of DHSs can not immediately be assigned to detectable gene transcriptional changes (Fig 5A) and yet may represent a genomic landscape of functional interest (see below).

Notably, STRING-db and literature analysis of the overlapping gene loci highlight evidence for functional and/or biochemical interactions with Wnt/β-catenin signaling functions (e.g., *Cdh1*, *Klf9*, *Src*, *Tgfbr2*, *Ddah1*, and *Ank3*), a pathway strongly reported to mechanistically contribute to PB-mediated liver tumor promotion (Aydinlik et al, 2001; Rignall et al, 2011; Groll et al, 2016), overall suggesting functional relevance of this concordant Δ-strain landscape. To verify that DHS and RNA changes (Fig 4) represent functionally relevant changes, we next evaluated protein expression levels of a subset of candidates relevant to Wnt/β-catenin signaling and for which well-characterized antibodies are available. We leveraged matching liver tissue samples and ran semiquantitative Western blot analyses of both Cdh1 and Src, confirming an increase in protein levels in PB-treated B6C3F1 mice (Fig 4C and F). These data highlight that a subset of PB-mediated hepatic responses of functional relevance can be discerned at both RNA and chromatin accessibility levels. Chromatin accessibility changes, akin to PB-mediated DNA (hydroxy)methylation effects (Thomson et al, 2013), may thus represent potential early biomarker signatures for non-genotoxic carcinogen exposure.

### Exploring transcriptional regulators of the noncoding, strain-selective Δ-DHS landscape

The vast majority of strain-selective changes in DHSs after PB treatment do not account for proximal changes in gene expression but may still represent important gene regulatory elements underlying mouse strain differences in tumor promotion sensitivity (Fig 5A). The *cis*-acting gene regulatory element landscape is in particular strongly enriched for transcription factor–binding sites (TFBSs) and represents critical genome regulatory information (Consortium, 2012; Shen et al, 2012; Thurman et al, 2012; Johanson et al, 2018). We, therefore, performed TF motif enrichment analysis individually on the up- or down-regulated PB-responsive strain-selective Δ-DHS landscape for each mouse strain. Because TF-binding motifs frequently overlap between different TFs, we used the root motifs from the motif clustering provided in the JASPAR database in our analysis. To strengthen the robustness of the detected motifs further, we also performed de novo motif analysis and overlapped the results with the motifs detected in the enrichment analysis. The resulting enriched motif clusters with associated similar de novo motifs are shown in Fig 5B and Table S5.

Table S5 Strain-specific transcription factor regulatory pathway effects identified by motif enrichment analysis. EnrichedMotifClusters: For each DHS group, the table lists the known JASPAR motif clusters identified as enriched in the Homer analysis, for which Homer also identified a highly similar de novo motif. Statistics on the enrichment test from Homer are given, including the *P*-value and q-value, the observed fold-change of enrichment, the percent of the target set, and the percent of the background DHS set that show the motif. Finally, the enrichment statistics from Homer for the best matching de novo motif are listed. Genes corresponding to the motif cluster are listed in the final column. Only genes with at least 100 RNA-seq read counts summed across all samples are shown, ordered by average expression across samples. cluster1_delta_peak_B6C3F1_up: GO biological pathway analysis of predicted TF target sites in the respective Δ-DHS group. cluster3_delta_peak_C57BL6_up: GO biological pathway analysis of predicted TF target sites in the respective Δ-DHS group. cluster11_delta_peak_B6C3F1_up: GO biological pathway analysis of predicted TF target sites in the respective Δ-DHS group. cluster12_delta_peak_B6C3F1_up: GO biological pathway analysis of predicted TF target sites in the respective Δ-DHS group. cluster19_delta_peak_B6C3F1_dow: GO biological pathway analysis of predicted TF target sites in the respective Δ-DHS group. cluster32_delta_peak_B6C3F1_dow: GO biological pathway analysis of predicted TF target sites in the respective Δ-DHS group. cluster66_delta_peak_B6C3F1_up: GO biological pathway analysis of predicted TF target sites in the respective Δ-DHS group. cluster10_delta_peak_B6C3F1_up: GO biological pathway analysis of predicted TF target sites in the respective Δ-DHS group. cluster10_delta_peak_C57BL6_dow: GO biological pathway analysis of predicted TF target sites in the respective Δ-DHS group.

In this analysis, cluster 32 (Stat family members, Bcl6, and Bcl6b) and cluster 19 (Nfat family members and Rbpj), both based on down-regulated B6C3F1 DHS landscape gave the strongest enrichment (low *P*-values, q-values, high FC enrichments, and several good de novo motif predictions). Two additional clusters were identified albeit less robustly using up-regulated B6C3F1-unique DHSs: cluster 11 (Smad family members, Tbx family members, and Srebf1/2) and cluster 12 (Fox family members). Only one cluster was weakly identified from C57BL/6J up-regulated DHS landscape (includes Jun/Fos, Bach, and other TF motifs), despite a similar count of strain-specific open chromatin changes (Fig 2A).

To evaluate the biological function of the different TF motifs clusters, we first predicted gene targets of Δ-strain DHS landscape for each of the enriched TF clusters and ran GO term enrichment analysis (Fig 5C and Table S5). Overall, this analysis highlighted a number of biological functions of potential relevance to early tumor development that were predominantly observed in the B6C3F1 strain after treatment with PB (e.g., change in nuclear functions, metabolic changes, epithelial to mesenchymal transition, cell–matrix adhesion/organization, etc.).

To help filter and assess functional relevance of the identified TF motifs, we next examined individual TFs using different knowledge-based sources such as STRING-db, GeneCards database, and literature assessments. We systematically interrogated the prior knowledge towards relevance of the TFs in PB response, CAR activation, Wnt signaling, and/or hepatocarcinoma.

Stat proteins are a family of seven TFs that form part of the JAK-STAT signaling cascade. Significant evidence support functional implication of Stat family members in hepatocarcinoma and cross-interactions with some of the core genes and pathways identified from PB-mediated changes in RNA or open chromatin effects (such as Src and TGF-β), ultimately consistent with the early tumor promotion relevance of this cluster of TF motifs in B6C3F1 mice (see the Discussion section). Notably, Bcl6, in the same cluster 32, is strongly reported to functionally interact with Stat family members, including through the regulation of interferon-γ (Madapura et al, 2017), representing one of the enriched GO biological pathway for this cluster (Fig 5C and Table S5). Consistent with its decreased motif activity, the tumor suppressor Bcl6B was also previously identified to inhibit HCC metastases in vitro and in vivo (Wang et al, 2014). Likewise, and also consistent with decreased motif activity, NFAT family members such as NFTAc1 are reported as tumor suppressor in HCC, through inducing tumor cell apoptosis (Xu et al, 2018). Examining the up-regulated DHS-associated TF motifs (clusters 11 and 12) also highlights core functional and biochemical associations in liver cell proliferation control and HCC of TGFβ/Smad proteins and pathway (Yoshida et al, 2014) or of selected Fox TF members. For example, Foxo1 is known to be under the regulatory control of CAR (Kazantseva et al, 2014) and a direct interactor of β-catenin in certain conditions (Liu et al, 2011).

Overall, these analyses highlight TF families that may play a role in determining mouse strain–specific liver tumorigenic responses to PB (Fig 5D and E). Despite an overall equivalent number of Δ-DHS signatures in both strains, the TF motifs are particularly enriched in PB-treated B6C3F1. Notably, some of these TFs have previously been associated with liver tumor promotion (including the Wnt/β-catenin signaling pathway), whereas others represent potentially novel regulatory factors for this hepatic phenotype.

## Discussion

Gene regulation depends on the coordinated interplay of local and long-range chromatin interactions within the three-dimensional space of the nucleus. In particular, it has been noted that noncoding regions of the genome contain *cis*-acting gene regulatory elements (e.g., enhancers) that determine cell type–specific functions through interactions with TFs. Importantly, the perturbation of enhancers has been associated with tumor development (Davie et al, 2015; Sur & Taipale, 2016). Enhancers are characterized by cell type–specific open chromatin structures that enable access for specific TFs and can be efficiently mapped using nuclease-based assays coupled with deep sequencing. In this study, we have exploited well-characterized mouse strain–specific differences in sensitivity to PB-mediated liver tumor promotion (Table S1) to investigate the underlying molecular mechanisms that could account for differences in phenotypic outcome. We have focused on liver tissue–based genome-wide profiling of chromatin accessibility (DNase-seq) and gene expression (RNA-seq) to characterize early molecular perturbations of *cis*-acting gene regulatory elements after exposure to tumor-promoting doses of PB. We identify quantitative differences in PB-induced hepatic molecular pathways and gene regulatory networks between two mouse strains that may account for differences in their sensitivity to liver tumor promotion. These observations also provide an entry point for investigating potential links between mouse strain–specific genetic variation and liver-specific gene regulatory landscapes after PB exposure.

Our molecular analysis on total liver reveals PB-induced chromatin accessibility and transcriptional changes that are either common to resistant (C57BL/6J) versus sensitive (B6C3F1) mouse strains or predominantly strain selective. Most chromatin accessibility changes take place at distal regulatory regions consistent with an important role of *cis*-acting gene regulatory elements in mediating early hepatic molecular responses to PB. We interrogated the function of the distal regulatory regions by mapping to the most proximal gene annotation (based on the position of the gene TSS) and by investigating TF motifs associated with changes in chromatin accessibility. These complementary approaches were integrated with gene expression and GO-based pathway analyses to highlight candidate groups of genes and/or molecular pathways associated with the early stage of PB-mediated non-genotoxic hepatocarcinogenesis. Importantly, these analyses highlighted quantitative differences between sensitive and resistant mouse strains in molecular pathways known to be associated with PB-mediated liver tumor promotion including Wnt/β-catenin signaling.

In addition, we also identified candidate TF regulators via PB-mediated changes in chromatin accessibility that may represent novel early biomarkers signatures of PB-mediated hepatocarcinogenesis. Our observation of down-regulation of DHSs containing Stat/Bcl6 family TF motifs (cluster 32) is suggestive of reduced Stat signaling activity in PB-treated B6C3F1 mice. There is significant evidence for a role of RAS and JAK/STAT pathway activation in HCC and therapeutic modulation of these pathways for the treatment of human liver cancer is being actively explored (Calvisi et al, 2006). Recent studies have also elucidated specific functions for JAK and STAT protein family members in Non Alcoholic Steato Hepatitis (NASH) and HCC (Grohmann et al, 2018; Kaltenecker et al, 2018). Genetic disruption of Stat5 activity in a mouse model for liver fibrosis was associated with elevated TGF-β levels and enhanced growth hormone-induced Stat3 activity, ultimately contributing to the development of chronic liver disease (Hosui et al, 2009). Furthermore, STAT3 has been reported to confer hepatoprotective or oncogenic functions depending on the extent and duration of additional stressors, including inflammation (Wang et al, 2011). Consistent with our observations of reduced Stat pathway–associated TF activity in PB-treated mice, Stat5b expression levels were reported to be suppressed in the livers of mice treated with CAR activators, including PB (Oshida et al, 2016). Interestingly, we also observed up-regulation of DHSs having TF motifs (cluster 11) that included Smad family TFs in PB-treated B6C3F1 mice, implying a potential increase in Smad activity. Together, our data would be consistent with a previous report of TGF-β induced SMAD signaling in hepatoma cells (Buenemann et al, 2001), and the potential for upstream regulation of TGF-β by Stat5 (Hosui et al, 2009).

The down-regulation of DHSs containing Bcl6 and Bcl6b TF motifs (cluster 32) implies a reduction in Bcl6-mediated signaling in PB-mediated B6C3F1 mice and is consistent with the low levels of BCL6B expression that have been previously reported as a potential prognostic biomarker for HCC (Wang et al, 2015b). Interestingly, we previously reported Bcl6 as a candidate target of Zfp161 TF motif, whose hepatic activity was computationally inferred to be decreased after PB treatment of mice and hypothesized to participate in the regulation of quiescent hepatocyte G0–G1 transitions during both early and late stages of PB-mediated tumorigenesis (Luisier et al, 2014b).

The identification of TF motifs associated with the Fox family TFs (cluster 12) in up-regulated DHSs is suggestive of increased Fox-mediated signaling in PB-treated B6C3F1 mice. The Forkhead box (FOX) proteins are a family of TFs that respond to a wide range of external stimuli and regulate diverse biological processes both during development and throughout adult life (Golson & Kaestner, 2016). Aberrant activity of Fox family members Foxa1 and Foxa2 was previously reported as central component of sexual dimorphism of mouse HCC (Li et al, 2012). Likewise, the FOXQ1/NDRG1 axis was shown to exacerbate HCC initiation via enhancing cross talk between fibroblasts and tumor cells (Luo et al, 2018), and FOXO1 was reported to contribute to HCC through a different mode of action (Dong et al, 2017; Jia et al, 2018). Notably, FoxO1 was reported to directly cross talk with CAR to regulate p21 expression and cell proliferation (Kazantseva et al, 2014). Foxp1 was also reported as a candidate hepatic target for the Zfp161 TF motif whose hepatic activity was computationally inferred to be decreased after PB treatment of mice (Luisier et al, 2014b).

In addition to highlighting novel TF motifs and pathways, we also identified minimal but significant changes in hepatic chromatin accessibility in PB-treated B6C3F1 mice within a gene regulatory element (an IG differentially methylated region [IG-DMR]) that regulates the *Dlk1-Dio3* imprinted gene cluster. PB was previously reported to progressively up-regulate the expression of noncoding RNAs (including *Meg3*) encoded by the *Dlk1-Dio3* locus in a CAR and β-catenin–dependent manner (Lempiainen et al, 2013). PB-mediated up-regulation of *Meg3* localises to perivenous hepatocytes that have been associated with Wnt-signaling dependent stem cell–like properties (Wang et al, 2015a). *Meg3* expression has also been associated with mouse stem cell pluripotency (Liu et al, 2010; Stadtfeld et al, 2010) and a subset of human HCCs (Luk et al, 2011), suggesting that CAR activators such as PB may drive dedifferentiation of adult hepatocytes towards a stem cell–like state during the early stages of hepatocarcinogenesis. Interestingly, a hepatocarcinogenesis sensitivity locus *Hcs3* that was originally identified using mouse backcrosses and linkage analysis (Dragani et al, 1991; Gariboldi et al, 1993) maps within a few megabases of the *Dlk1-Dio3* cluster (Drinkwater & Ginsler, 1986; Lempiainen et al, 2013). Our data suggest a model in which PB-mediated transcriptional responses from this epigenetically imprinted gene cluster may be enhanced via increased chromatin accessibility that is mediated by B6C3F1 strain-selective genetic factors.

The potential contributions of PB-mediated changes in hepatic *cis*-acting gene regulatory elements and TFs to tumor promotion in B6C3F1 mice will require further biochemical and functional investigations. Nevertheless, our study highlights several candidate TFs that may act as co-effectors during the early stages of PB-mediated tumor promotion effects in susceptible mouse strains (Fig 5D). Most chromatin accessibility changes take place distally from gene promoters at putative enhancers. Enhancers are bound by regulatory TFs and serve as integrators of intracellular and extracellular signaling pathways to generate cell type–specific transcriptional responses (Shlyueva et al, 2014). The functional binding of regulatory TFs at *cis*-acting gene regulatory elements such as enhancers orchestrates long-range gene regulatory interactions within the three-dimensional space of the nucleus, enabling cell type–specific and spatiotemporal control of gene expression patterns which drive cell identity and function (Thurman et al, 2012; Jin et al, 2013; Zhang et al, 2013). Importantly, distally bound TFs can also regulate genome functions underlying cell identity through mechanisms that are independent of transcription (Johanson et al, 2018). Thus, the characterization of xenobiotic-induced perturbations of accessible chromatin landscapes and associated TF networks holds great potential for the identification of pathophysiologic mechanisms.

We recognize that the characterization of xenobiotic-mediated tissue-specific molecular responses within tissue derived from in vivo model systems is associated with some significant limitations. In particular, determining the specific cell types associated with molecular responses can be challenging. Multiple biological pathways can dynamically respond within and across cell types, and thus, a precise linkage of molecular effects to cellular phenotypes requires follow-up investigations (e.g., single-cell resolution molecular profiling coupled with pharmacologic and/or genetic modulation of pathway components). The functional annotation of long-range gene regulatory interactions (e.g., enhancers, transcriptional factor interactions, and nuclear organization) will require follow-up locus-/pathway-specific tools and functional analyses, including the direct biochemical mapping of inferred TF occupancy by chromatin immunoprecipitation. Likewise, future work will be necessary to investigate local and global genetic variations in context of strain-selective differences in chromatin accessibility and TF regulation.

The epigenomic landscape is strongly responsive to environmental conditions, including exposure to xenobiotics such as chemicals and pharmaceuticals (Jirtle & Skinner, 2007; Feil & Fraga, 2012) and, thus, represents an important molecular space for gaining potential insight into pharmacologic and toxicologic responses to xenobiotics (Szyf, 2007; Lewis et al, 2017; Israel et al, 2018). This study highlights the functional and toxicological relevance of hepatic epigenomic responses in a well-characterized rodent model for drug-induced non-genotoxic carcinogenesis. The mapping of mouse strain–selective pertubations of *cis*-acting regulatory elements (cistrome) provides novel TF–based insights into the molecular basis for susceptibility to PB-induced rodent liver tumor promotion (Fig 5E). These observations are also consistent with recently reported links between genetic variation and tissue-specific epigenetic perturbations after toxicant exposures (Lewis et al, 2017; Israel et al, 2018). Thus, the integration of genetic, epigenomic, and transcriptomic data together with phenotypic endpoints has the potential to enhance mechanism-based safety assessment of both chemicals and pharmaceuticals. In addition, integrated epigenomic and transcriptomic profiling of tissue-specific responses to xenobiotics may also provide a valuable resource for exploring gene regulatory networks underlying broader pathophysiologic processes.

## Materials and Methods

### Ethics statement

In vivo rodent studies were performed in conformity with the Swiss Animal Welfare Law (specifically under the animal licenses No. 2345 by Kantonales Veterinaramt Basel-Stadt [Cantonal Veterinary Office, Basel]).

### Animal treatment and sample preparation

As previously reported in (Lempiainen et al, 2013; Luisier et al, 2014a), male 9–11-wk-old C57BL/6J mice were obtained from Taconic. Animals were allowed to acclimatize for at least 5 d before being randomly divided into two treatment groups of five animals each. 0.05% (wt/vol) PB (Hänseler AG) was administered to one group through ad libitum access to drinking water for either 1, 7, 14, 28 or 91 d. Male B6C3F1/Ctrl (C57BL/6 ♀ × C3H/He ♂) mice 4–5 wk old were obtained from Charles River Laboratories. Animals were allowed to acclimatize for at least 5 d before being randomly divided into two treatment groups of five animals each. 0.05% (wt/vol) PB (Sigma-Aldrich) was administered to one group through ad libitum access to drinking water for 91 d. Individual mice from both studies were checked daily for activity and behavior and sacrificed on the last day of dosing. Livers were removed before freezing in liquid nitrogen and −80°C storage.

### Nuclei preparation, DNase treatment, and DNA purification

DNase I assay was performed as previously described by Ling and Waxman (Ling and Waxman 2013a, 2013b) with minor modifications. 100–150 mg of frozen liver tissue was thawed on ice for 5 min and then homogenized in 1 ml ice-cold nuclear homogenization buffer (NEHB): 10 mM Hepes-KOH, pH 8.0, 25 mM KCl, 1 mM EDTA, 2 M sucrose, 10% glycerol, 0.15 mM spermine, 0.5 mM spermidine, 10 mM NaF, 1 mM orthovanadate, 1 mM PMSF, 0.5 mM DTT, and 1× protease inhibitor cocktail; using a 7-ml Dounce tissue homogenizer. Nuclei were isolated by ultracentrifugation in an SW 40 Ti rotor, overlying the homogenized tissue on top of 5 ml NEHB in a 14-ml Thinwall Ultra-Clear 14 × 95-mm tube (cat. no. 344060; Beckman Coulter). Centrifugation was performed at 66,000*g* for 1 h at 4°C. Isolated nuclei were rinsed three times in 1 ml ice-cold buffer A: 15 mM Tris–HCl, pH 8.0, 15 mM NaCl, 60 mM KCl, 1 mM EDTA, 0.5 mM EGTA, 0.5 mM spermidine, and 0.3 mM spermine. The pelleted nuclei were suspended in digestion buffer (nine volumes of buffer A and one volume of 10X DNase I digestion buffer [60 mM CaCl_2_, 750 mM NaCl]) by using a Dounce homogenizer, counted using a hemacytometer after 100-fold dilution in PBS, and then resuspended in final aliquots of 250 μl containing ∼3.3 × 10^6^ nuclei each. DNase I digestion was carried out at 37°C for 3 min using a final concentration of 50 U/ml of RQ1 RNase-free DNase I (cat. no. M6101; Promega). We digested 10^7^ nuclei per mouse. DNase digestion was stopped using 300 μl of stop buffer: 50 mM Tris–HCl, pH 8.0, 100 mM NaCl, 0.1% SDS, 100 mM EDTA, 0.5 mM spermidine, and 0.3 mM spermine. DNA purification was performed at 55°C overnight using 10 μl of 10 mg/ml proteinase K. RNA digestion was carried out at 37°C for 30 min using 5 μl of 10 mg/ml RNase A. DNA from different aliquots deriving from the same animal were pooled and subsequently extracted using an equal volume of phenol–chloroform–isoamyl alcohol and then one volume of chloroform–isoamyl alcohol. The solution recovered from the phenolic extraction was adjusted with 5 M NaCl to give a final concentration of 0.8 M NaCl and then DNA fractionation was performed in a SW 40 Ti rotor, overlying the adjusted solution on top of 9 ml of a sucrose step gradient (40%, 35%, 30%, 25%, 20%, 17.5%, 15%, 12.5%, and 10% solutions in 1× centrifugation buffer: 20 mM Tris–HCl, pH 8.0, 5 mM EDTA, and 1 mM NaCl). Centrifugation was performed at 78,000*g* in a 14 ml Thinwall Ultra-Clear 14 × 95 mm tube for 24 h at 20°C setting acceleration and deceleration parameters at five. After ultracentrifugation, the first 3-ml fraction was recovered and purified over a MinElute Purification Column (QIAGEN) and eluted in 13 μl of provided Elution Buffer.

### DNase I library preparation and sequencing

DNase I digests the entire genome with a strong preference for regions that are devoid of nucleosomes and unoccupied by TF. Sub-nucleosomal fragments (<145 bp) are enriched for nucleosome-free and TF bound regions, which can be further analyzed to obtain important insights into the functional genomic landscape (Vierstra et al, 2014). We, therefore, size-selected the low molecular fraction of the DNase-seq libraries and performed 76-bp paired-end (PE) sequencing, obtaining an average of 43 million fragments per sample (Table S6). Specifically, next generation sequencing libraries were prepared with the NuGEN Ovation Ultralow System V2 with A-tailing (TECAN) from 3 to 20 ng of input material. Inputs smaller or higher than 10 ng were amplified using 16 or 13 rounds of PCR, respectively. Up to nine libraries were pooled before size selection of the 40–250 bp fraction on a BluePippin BDF3010 3% DF Marker Q2 cassette or PippinHT BEF2010 2% DF Marker M1 cassette (both Sage Science) and PE 76-bp sequencing was performed on the HiSeq 2500 using v4 reagents (Illumina).

Table S6 Sequencing statistics for each DNase-seq sample. Total number of fragments is based on all fragments after sequencing and de-multiplexing. The filtered number of fragments are derived from properly paired reads in the pre-processed bam files. The average fragment size is estimated from the insert sizes between read pairs stored in the pre-processed bam files.

### Genome imputation for B6C3F1

To mitigate the impact of strain differences in read mapping and assess chromatin variation across inbred mice (Hosseini et al, 2013), we generated strain-specific reference sequences using known Single Nucleotide Polymorphisms (SNPs) from the Mouse Genomes Project (Keane et al, 2011; Doran et al, 2016). We used the mm10 reference genome (C57BL/6J strain) as reference for the C57BL/6J samples, subselecting chromosomes chr1-19, X and Y using SAMtools faidx v0.1.19 (Li et al, 2009) for analysis. To impute the genome of B6C3F1, we downloaded the SNPs of the parental strain C3H/HeJ from the Mouse Genomes Project (C3H_HeJ.mgp.v5.snps.dbSNP142.vcf). After sorting using VCFtools v0.1.14 (Danecek et al, 2011), we introduced the C3H/HeJ-specific SNPs into the mm10 genome using the FastaAlternateReferenceMaker from the Genome Analysis Toolkit (McKenna et al, 2010). We generated index files with SAMtools faidx. Finally, to confirm the expected genotypes for each strain, we compared chromosomal sizes from mm10, C57BL/6J, and C3H/HeJ and manually inspected several loci with the Integrative Genomics Viewer v2.3 (Robinson et al, 2011; Thorvaldsdottir et al, 2013).

### DNase-seq data pre-processing

Libraries were demultiplexed using bcl2fastq version 2.17. Illumina adaptor sequences at the end of the reads were removed using Cutadapt v1.8 and resulting reads with a minimum length of 30 bp were retained. All samples were aligned to the mm10 reference genome using Bowtie2 v2.1.0 (Langmead & Salzberg, 2012) with the following parameters: -I 50 -X 400 --fr --no-mixed --no-discordant. Binary Alignment Map (Bam) files were created from sam files and coordinate sorted using SAMtools. PCR and optical sequencing duplicates were flagged using Picard Tools’ MarkDuplicates v1.107(1667). Only properly paired, unique reads with a map quality score above 12 were retained after filtering using SAMtools. Bam files of the same biological replicate from multiple sequencing runs were merged and again filtered for PCR or optical sequencing duplicates.

In addition, we mapped the B6C3F1 samples to the imputed C3H/HeJ genome and compared the results of the alignments with the two different reference genomes using bamUtil diff with options --mapQual--mate. Under these conditions, no differences were detected in common read pairs, and less than 50–100 read pairs resulted to be unique for one or the other alignment. Hence, we decided to use the alignment to the mm10 reference for all downstream analysis.

### Re-mapping of the encyclopedia of DNA elements (ENCODE) liver data

The mouse DNase-seq and ChIP-seq data were integrated from mouse ENCODE (GSE37074 and GSE31039, respectively). FASTQ files were retrieved from NCBI SRA (https://www.ncbi.nlm.nih.gov/sra) and processed with the same pipeline above.

### DHS calling and differential analysis on DNase-seq data

DHSs were identified and *bedGraphs* were created applying a q-value cutoff (−*q*) of 0.01 using MACS2 v2.1.1.20160309 (Zhang et al, 2008). Differential peak analysis was performed in R 3.2.3 using the Bioconductor package *DiffBind* v1.16.3 (Ross-Innes et al, 2012). Consensus peaks for each condition were created using *dba.counts* requiring that at least three samples show a significant peak in the same genomic window (minOverlap = 3). The *DBA_EDGER* method was used to call differential peaks including treatment and strain in the contrast. Regions showing an absolute log2 fold-change |log2 FC| ≥ 0.58 between selected contrasts and a FDR ≤ 0.01 (Benjamini–Hochberg) are called differential.

### DHS coverage profile

Coverage profiles were generated from the bam files by counting reads in 50-bp bins within the 5-kb region centered at the midpoint of each consensus DHS. The bamProfile function in the bamsignals (v1.12.1) R package was used setting the PE flag to “ignore.” The profiles were scaled to the average across samples of the total number of reads falling into the consensus DHSs.

### Overlapping DHSs with gene annotation

To assign DHSs to promoters, intra-, or intergenic elements, the function assignChromosomeRegion in the ChIPpeakAnno v3.16.1 R package (Zhu et al, 2010; Zhu, 2013) was used on the UCSC known gene annotation (https://genome.ucsc.edu/). DHSs assigned to the categories *fiveUTRs*, *threeUTRs*, *Exons*, or *Introns *were called intragenic, whereas DHSs assigned to the categories *Intergenic. Region* and *Immediate Downstream* were called Intergenic (categories as per R package).

### Browser track visualization of DNase-seq data

For visualization of individual loci, edgeR-normalized DNase-seq tracks were produced converting BAM files into BedGraphs using BEDtools 2.24.0 genomecov with *–bg* and –scale parameters (Quinlan, 2014). Sizes of the libraries were obtained from the dba.analyse object (bFullLibrarySize = TRUE). Scaling factors were calculated by dividing the minimum library count by the size of each individual library. Bigwig files were compiled from normalized BedGraphs using bedGraphToBigWig v4. Coverage of individual loci were plotted in R3.5.0 using the wiggleplotr 1.6.1 R package.

### Assigning DHSs to closest TSS

DHSs were mapped to the closest TSS using the chipenrich v2.6.1 R package (Welch et al, 2014) and the predefined locus definition nearest_tss for the mm10 genome.

### STRING-db sub-network enrichment analysis

The STRINGdb v1.22.0 R package (Franceschini et al, 2013) was used to identify enriched protein–protein interaction sub-networks among predefined gene sets. The complete network (version 10) for the *Mus Musculus* (10,090) organism was selected and only gene symbol that mapped to STRINGdb IDs were retained. Sub-networks were identified using the Walktrap community-finding algorithm implemented in igraph v.1.2.4.

### Total RNA extraction and sequencing

25–50 mg of frozen liver material was used for total RNA extraction using 1 ml TRIzol reagent (cat. no. 15596026; Thermo Fisher Scientific). Samples were homogenized using a MagNA Lyser homogenizer (Roche) using two runs of 30 s at 6,500 rpm. Aliquots of ∼600 μl of homogenized material were transferred into pre-spun 2-ml Phase Lock Gel-Heavy tubes (5Prime) and incubated for 5 min at room temperature. 100 μl chloroform–isoamyl alcohol was added to each aliquot. The tubes were shaken vigorously for 15 s and incubated for 2 min. The tubes were centrifuged at 12,000*g* for 10 min at 4°C allowing phase separation. Sample-matching upper aqueous phases were pooled into new pre-spun 2-ml Phase Lock Gel-Heavy tubes and additional 200 μl chloroform–isoamyl alcohol was used to perform a second phase separation. Aqueous phases were then measured and transferred into fresh tubes where RNA precipitation with 0.5 ml isopropyl alcohol was performed. After 10-min incubation, the samples were centrifuged at 12,000*g* for 10 min at 4°C. The precipitated RNA was washed once by using 1 ml 75% ethanol before resuspension in EB buffer (QIAGEN). The samples were NanoDrop-quantified and the RNA integrity number measured using an Agilent RNA 6000 Nano Kit (cat. no. 5067-1511; Agilent Technologies) on a BioAnalyzer instrument. Samples with RNA integrity number above 8 were used for sequencing. Illumina libraries were prepared with the TruSeq Stranded Total RNA Sample Preparation kit with Ribo-Zero Gold from 100 or 150 ng of input RNA using 13 or 12 rounds of PCR for amplification, respectively. We generated PE 76-bp reads on a HiSeq 2500 using v4 reagents (Illumina).

### RNA-seq data processing

Libraries were demultiplexed and then passed to the STAR v2.4.0f1 aligner (Dobin et al, 2013) to map reads to the UCSC reference genome mm10. The resulting bam files were sorted using SAMtools. Bam files of the same biological replicate from different sequencing runs were merged and filtered using SAMtools with options -q 255 and -F 512. To quantify gene expression, reads overlapping genes in the UCSC RefSeq gene annotation were counted using the summarizeOverlap function (with parameters mode = “Union,” singleEnd = FALSE, ignore.strand = FALSE, and fragments = TRUE) of the GenomicAlignments (v1.6.3) R package.

### Differential gene expression analysis on RNA-seq data

EdgeR (v.3.12.1) (Robinson et al, 2010; McCarthy et al, 2012) was used to perform differential expression analysis on the count matrix. Only genes that show CPM ≥ 0.4 in at least three samples per treatment-strain group in at least one group were retrained for analysis. Normalization factors to scale for library size were calculated using the trimmed mean of M values (TMM) method. The design is based on assigning each sample to a treatment-strain group. The glmQLFit and glmQLFTest methods were used to fit a quasi-likelihood negative binomial generalized log-linear model and to perform gene-wise tests for differential expression. Differentially expressed genes were identified as genes showing at least |log2 FC| ≥ 0.58 between selected contrasts and a FDR ≤ 0.01.

### TFBS enrichment

We searched the different groups of DHSs for enrichment of TF binding sites using the HOMER software (Heinz et al, 2010). We performed both, de novo motif analysis and enrichment analysis of known TF motifs and overlapped the results to strengthen the robustness of the identified motifs.

For the enrichment analysis, we obtained known TF binding motifs from the JASPAR database (Khan et al, 2018). Because motifs of different TFs can be very similar, we used the root motifs from the TF motif clustering provided in the JASPAR database as input for HOMER. The score cutoffs to call binding sites were optimized for each root motif by running HOMER’s findMotifs on all DHSs and taking the score that defined the 88% quantile based on the resulting score distribution. Finally, we ran HOMER’s findMotif on the individual DHS groups providing the fasta files of all DHS regions for background parameter calculation and obtained enrichment and de novo analysis results. The Regulatory Sequence Analysis Tools (RSAT) (Nguyen et al, 2018) compare-matrices tool with default values was used to correlate de novo analysis-derived TF motifs with the known root motifs. We overlapped the results by assigning each known root motif the de novo motif with highest correlation.

### Protein extraction, concentration measurement, and immunoblotting

Liver tissue was dissociated in ice-cold Radioimmunoprecipitation assay (RIPA) buffer (89900; Pierce) complemented with proteases and phosphatases inhibitors (P8340, P2850, and P5726-1/100 each; Sigma-Aldrich) using the Covaris ultrasound homogenizer. Liver lysates were centrifuged (15 min, 16,000*g*, 4°C) and supernatants collected and stored at −80°C. SDS–PAGE samples were prepared in NuPAGE 4× buffer (NP0007; Invitrogen) and 10× reducing agent (NP0004; Invitrogen) or in 6× loading buffer (60 mM Tris, pH 6.8, 2% SDS, 9.8% glycerol, bromophenol blue, 0.6 mM DTT). Samples were heated (10 min, 70°C), centrifuged (3 min, 14,000*g*), and resolved on Invitrogen NuPAGE Bis-Tris gels (4–12% or 8% gel, MES running buffer, 160 V, 1 h). The gels were transferred to nitrocellulose membranes (20 V, 7 min) using the I-Blot system (#IB1001EU; Invitrogen). Membranes were blocked for 1 h in Odyssey Blocking Buffer (LI-COR 927-40000)/PBS (1:1) and subsequently incubated overnight at 4°C with primary antibodies (anti-Src, CST2123, rabbit polyclonal IgG, 1:1,000; anti–E-Cadh, CST3195, rabbit polyclonal IgG, 1:1,000; anti-actin, A5060; Sigma-Aldrich, rabbit polyclonal IgG, 1:15,000) diluted in Odyssey Blocking Buffer/PBS-Tween 0.1% (1:1). After washing three times with PBS-Tween 0.1%, the membranes were incubated for 1 h with secondary antibodies labelled with IRdyes diluted in Odyssey Blocking Buffer/PBS-Tween 0.1% (1:1). The membranes were washed (three times with PBS-Tween 0.1% and three times with PBS) and dried before scanning using the Odyssey Infrared Imaging System (LI-COR). Integrated intensities of the protein bands of each target were quantified in their respective fluorescent channels using Odyssey software (background subtraction top/bottom of quantified band). The integrated intensity for Src and E-Cadh were normalized to the integrated intensity for actin and displayed as % of control.

**Table:** List of software and databases.

Software/Database/R package	Reference/Web link
bcl2fastq	https://support.illumina.com/sequencing/sequencing_software/bcl2fastq-conversion-software.html
Picard	http://broadinstitute.github.io/picard/
bamUtil	https://genome.sph.umich.edu/wiki/BamUtil
bamsignals	http://bioconductor.org/packages/release/bioc/html/bamsignals.html
bedGraphToBigWig	http://hgdownload.soe.ucsc.edu/admin/exe/linux.x86_64/
wiggleplotr	http://bioconductor.org/packages/release/bioc/html/wiggleplotr.html
igraph	https://igraph.org
GenomicAlignments	http://bioconductor.org/packages/release/bioc/html/GenomicAlignments.html
STRING database	http://string-db.org/
JASPAR motif database	http://jaspar.genereg.net/
GeneCard database	https://www.genecards.org/
Cutadapt	https://doi.org/10.14806/ej.17.1.200

## Data Deposition

All data generated in this manuscript are included in the NCBI GEO under the accession number GSE131344.

## Supplementary Material

Reviewer comments

## References

[bib1] Allis CD, Jenuwein T (2016) The molecular hallmarks of epigenetic control. Nat Rev Genet 17: 487–500. 10.1038/nrg.2016.59 27346641

[bib2] Aydinlik H, Nguyen TD, Moennikes O, Buchmann A, Schwarz M (2001) Selective pressure during tumor promotion by phenobarbital leads to clonal outgrowth of beta-catenin-mutated mouse liver tumors. Oncogene 20: 7812–7816. 10.1038/sj.onc.1204982 11753661

[bib3] Bachman AN, Phillips JM, Goodman JI (2006) Phenobarbital induces progressive patterns of GC-rich and gene-specific altered DNA methylation in the liver of tumor-prone B6C3F1 mice. Toxicol Sci 91: 393–405. 10.1093/toxsci/kfj155 16537655

[bib4] Becker FF (1982) Morphological classification of mouse liver tumors based on biological characteristics. Cancer Res 42: 3918–3923. 7104991

[bib5] Buenemann CL, Willy C, Buchmann A, Schmiechen A, Schwarz M (2001) Transforming growth factor-beta1-induced Smad signaling, cell-cycle arrest and apoptosis in hepatoma cells. Carcinogenesis 22: 447–452. 10.1093/carcin/22.3.447 11238185

[bib6] Calvisi DF, Ladu S, Factor VM, Thorgeirsson SS (2004) Activation of beta-catenin provides proliferative and invasive advantages in c-myc/TGF-alpha hepatocarcinogenesis promoted by phenobarbital. Carcinogenesis 25: 901–908. 10.1093/carcin/bgh083 14742323

[bib7] Calvisi DF, Ladu S, Gorden A, Farina M, Conner EA, Lee JS, Factor VM, Thorgeirsson SS (2006) Ubiquitous activation of Ras and Jak/Stat pathways in human HCC. Gastroenterology 130: 1117–1128. 10.1053/j.gastro.2006.01.006 16618406

[bib8] Consortium EP (2012) An integrated encyclopedia of DNA elements in the human genome. Nature 489: 57–74. 10.1038/nature11247 22955616 PMC3439153

[bib9] Corradin O, Saiakhova A, Akhtar-Zaidi B, Myeroff L, Willis J, Cowper-Sal lari R, Lupien M, Markowitz S, Scacheri PC (2014) Combinatorial effects of multiple enhancer variants in linkage disequilibrium dictate levels of gene expression to confer susceptibility to common traits. Genome Res 24: 1–13. 10.1101/gr.164079.113 24196873 PMC3875850

[bib10] Danecek P, Auton A, Abecasis G, Albers CA, Banks E, DePristo MA, Handsaker RE, Lunter G, Marth GT, Sherry ST, (2011) The variant call format and VCFtools. Bioinformatics 27: 2156–2158. 10.1093/bioinformatics/btr330 21653522 PMC3137218

[bib11] Davie K, Jacobs J, Atkins M, Potier D, Christiaens V, Halder G, Aerts S (2015) Discovery of transcription factors and regulatory regions driving in vivo tumor development by ATAC-seq and FAIRE-seq open chromatin profiling. PLoS Genet 11: e1004994 10.1371/journal.pgen.1004994 25679813 PMC4334524

[bib12] Ding ZY, Jin GN, Liang HF, Wang W, Chen WX, Datta PK, Zhang MZ, Zhang B, Chen XP (2013) Transforming growth factor beta induces expression of connective tissue growth factor in hepatic progenitor cells through Smad independent signaling. Cell Signal 25: 1981–1992. 10.1016/j.cellsig.2013.05.027 23727026

[bib13] Diwan BA, Rice JM, Ward JM (1986) Tumor-promoting activity of benzodiazepine tranquilizers, diazepam and oxazepam, in mouse liver. Carcinogenesis 7: 789–794. 10.1093/carcin/7.5.789 3698206

[bib14] Dobin A, Davis CA, Schlesinger F, Drenkow J, Zaleski C, Jha S, Batut P, Chaisson M, Gingeras TR (2013) STAR: Ultrafast universal RNA-seq aligner. Bioinformatics 29: 15–21. 10.1093/bioinformatics/bts635 23104886 PMC3530905

[bib15] Dong T, Zhang Y, Chen Y, Liu P, An T, Zhang J, Yang H, Zhu W, Yang X (2017) FOXO1 inhibits the invasion and metastasis of hepatocellular carcinoma by reversing ZEB2-induced epithelial-mesenchymal transition. Oncotarget 8: 1703–1713. 10.18632/oncotarget.13786 27924058 PMC5352090

[bib16] Doran AG, Wong K, Flint J, Adams DJ, Hunter KW, Keane TM (2016) Deep genome sequencing and variation analysis of 13 inbred mouse strains defines candidate phenotypic alleles, private variation and homozygous truncating mutations. Genome Biol 17: 167 10.1186/s13059-016-1024-y 27480531 PMC4968449

[bib17] Dragani TA (2010) Risk of HCC: Genetic heterogeneity and complex genetics. J Hepatol 52: 252–257. 10.1016/j.jhep.2009.11.015 20022654

[bib18] Dragani TA, Manenti G, Della Porta G (1991) Quantitative analysis of genetic susceptibility to liver and lung carcinogenesis in mice. Cancer Res 51: 6299–6303. 1933890

[bib19] Drinkwater NR, Ginsler JJ (1986) Genetic control of hepatocarcinogenesis in C57BL/6J and C3H/HeJ inbred mice. Carcinogenesis 7: 1701–1707. 10.1093/carcin/7.10.1701 3757172

[bib20] Farber E (1984) The multistep nature of cancer development. Cancer Res 44: 4217–4223. 6467183

[bib21] Feil R, Fraga MF (2012) Epigenetics and the environment: Emerging patterns and implications. Nat Rev Genet 13: 97–109. 10.1038/nrg3142 22215131

[bib22] Feinberg AP, Koldobskiy MA, Gondor A (2016) Epigenetic modulators, modifiers and mediators in cancer aetiology and progression. Nat Rev Genet 17: 284–299. 10.1038/nrg.2016.13 26972587 PMC4888057

[bib23] Franceschini A, Szklarczyk D, Frankild S, Kuhn M, Simonovic M, Roth A, Lin J, Minguez P, Bork P, von Mering C, (2013) STRING v9.1: Protein-protein interaction networks, with increased coverage and integration. Nucleic Acids Res 41: D808–D815. 10.1093/nar/gks1094 23203871 PMC3531103

[bib24] Gariboldi M, Manenti G, Canzian F, Falvella FS, Pierotti MA, Della Porta G, Binelli G, Dragani TA (1993) Chromosome mapping of murine susceptibility loci to liver carcinogenesis. Cancer Res 53: 209–211. 8417808

[bib25] Ghouri YA, Mian I, Rowe JH (2017) Review of hepatocellular carcinoma: Epidemiology, etiology, and carcinogenesis. J Carcinog 16: 1 10.4103/jcar.JCar_9_16 28694740 PMC5490340

[bib26] Goldsworthy TL, Fransson-Steen R (2002) Quantitation of the cancer process in C57BL/6J, B6C3F1 and C3H/HeJ mice. Toxicol Pathol 30: 97–105. 10.1080/01926230252824770 11890483

[bib27] Golson ML, Kaestner KH (2016) Fox transcription factors: From development to disease. Development 143: 4558–4570. 10.1242/dev.112672 27965437 PMC5201025

[bib28] Grohmann M, Wiede F, Dodd GT, Gurzov EN, Ooi GJ, Butt T, Rasmiena AA, Kaur S, Gulati T, Goh PK, (2018) Obesity drives STAT-1-dependent NASH and STAT-3-dependent HCC. Cell 175: 1289–1306.e20. 10.1016/j.cell.2018.09.053 30454647 PMC6242467

[bib29] Groll N, Petrikat T, Vetter S, Wenz C, Dengjel J, Gretzmeier C, Weiss F, Poetz O, Joos TO, Schwarz M, (2016) Inhibition of beta-catenin signaling by phenobarbital in hepatoma cells in vitro. Toxicology 370: 94–105. 10.1016/j.tox.2016.09.018 27693619

[bib30] Heindryckx F, Colle I, Van Vlierberghe H (2009) Experimental mouse models for hepatocellular carcinoma research. Int J Exp Pathol 90: 367–386. 10.1111/j.1365-2613.2009.00656.x 19659896 PMC2741148

[bib31] Heinz S, Benner C, Spann N, Bertolino E, Lin YC, Laslo P, Cheng JX, Murre C, Singh H, Glass CK (2010) Simple combinations of lineage-determining transcription factors prime cis-regulatory elements required for macrophage and B cell identities. Mol Cell 38: 576–589. 10.1016/j.molcel.2010.05.004 20513432 PMC2898526

[bib32] Hnisz D, Abraham BJ, Lee TI, Lau A, Saint-Andre V, Sigova AA, Hoke HA, Young RA (2013) Super-enhancers in the control of cell identity and disease. Cell 155: 934–947. 10.1016/j.cell.2013.09.053 24119843 PMC3841062

[bib33] Honkakoski P, Negishi M (1997) Characterization of a phenobarbital-responsive enhancer module in mouse P450 Cyp2b10 gene. J Biol Chem 272: 14943–14949. 10.1074/jbc.272.23.14943 9169466

[bib34] Hosseini M, Goodstadt L, Hughes JR, Kowalczyk MS, de Gobbi M, Otto GW, Copley RR, Mott R, Higgs DR, Flint J (2013) Causes and consequences of chromatin variation between inbred mice. PLoS Genet 9: e1003570 10.1371/journal.pgen.1003570 23785304 PMC3681629

[bib35] Hosui A, Kimura A, Yamaji D, Zhu BM, Na R, Hennighausen L (2009) Loss of STAT5 causes liver fibrosis and cancer development through increased TGF-{beta} and STAT3 activation. J Exp Med 206: 819–831. 10.1084/jem.20080003 19332876 PMC2715112

[bib36] Israel JW, Chappell GA, Simon JM, Pott S, Safi A, Lewis L, Cotney P, Boulos HS, Bodnar W, Lieb JD, (2018) Tissue- and strain-specific effects of a genotoxic carcinogen 1,3-butadiene on chromatin and transcription. Mamm Genome 29: 153–167. 10.1007/s00335-018-9739-6 29429127 PMC6095468

[bib37] Jensen LJ, Kuhn M, Stark M, Chaffron S, Creevey C, Muller J, Doerks T, Julien P, Roth A, Simonovic M, (2009) STRING 8--a global view on proteins and their functional interactions in 630 organisms. Nucleic Acids Res 37: D412–D416. 10.1093/nar/gkn760 18940858 PMC2686466

[bib38] Jia Y, French B, Tillman B, French S (2018) Different roles of FAT10, FOXO1, and ADRA2A in hepatocellular carcinoma tumorigenesis in patients with alcoholic steatohepatitis (ASH) vs non-alcoholic steatohepatitis (NASH). Exp Mol Pathol 105: 144–149. 10.1016/j.yexmp.2018.07.005 30009772 PMC6093215

[bib39] Jin F, Li Y, Dixon JR, Selvaraj S, Ye Z, Lee AY, Yen CA, Schmitt AD, Espinoza CA, Ren B (2013) A high-resolution map of the three-dimensional chromatin interactome in human cells. Nature 503: 290–294. 10.1038/nature12644 24141950 PMC3838900

[bib40] Jirtle RL, Skinner MK (2007) Environmental epigenomics and disease susceptibility. Nat Rev Genet 8: 253–262. 10.1038/nrg2045 17363974 PMC5940010

[bib41] Johanson TM, Lun ATL, Coughlan HD, Tan T, Smyth GK, Nutt SL, Allan RS (2018) Transcription-factor-mediated supervision of global genome architecture maintains B cell identity. Nat Immunol 19: 1257–1264. 10.1038/s41590-018-0234-8 30323344

[bib42] Kaltenecker D, Themanns M, Mueller KM, Spirk K, Suske T, Merkel O, Kenner L, Luis A, Kozlov A, Haybaeck J, (2018) Hepatic growth hormone - JAK2 - STAT5 signalling: Metabolic function, non-alcoholic fatty liver disease and hepatocellular carcinoma progression. Cytokine S1043–4666(18)30398–3. 10.1016/j.cyto.2018.10.010 30389231

[bib43] Kazantseva YA, Yarushkin AA, Pustylnyak VO (2014) CAR-mediated repression of Foxo1 transcriptional activity regulates the cell cycle inhibitor p21 in mouse livers. Toxicology 321: 73–79. 10.1016/j.tox.2014.04.003 24769335

[bib44] Keane TM, Goodstadt L, Danecek P, White MA, Wong K, Yalcin B, Heger A, Agam A, Slater G, Goodson M, (2011) Mouse genomic variation and its effect on phenotypes and gene regulation. Nature 477: 289–294. 10.1038/nature10413 21921910 PMC3276836

[bib45] Khan A, Fornes O, Stigliani A, Gheorghe M, Castro-Mondragon JA, van der Lee R, Bessy A, Cheneby J, Kulkarni SR, Tan G, (2018) JASPAR 2018: Update of the open-access database of transcription factor binding profiles and its web framework. Nucleic Acids Res 46: D260–D266. 10.1093/nar/gkx1188 29140473 PMC5753243

[bib46] Konno Y, Kamino H, Moore R, Lih F, Tomer KB, Zeldin DC, Goldstein JA, Negishi M (2010) The nuclear receptors constitutive active/androstane receptor and pregnane x receptor activate the Cyp2c55 gene in mouse liver. Drug Metab Dispos 38: 1177–1182. 10.1124/dmd.110.032334 20371638 PMC2908984

[bib47] Langmead B, Salzberg SL (2012) Fast gapped-read alignment with Bowtie 2. Nat Methods 9: 357–359. 10.1038/nmeth.1923 22388286 PMC3322381

[bib48] Lempiainen H, Couttet P, Bolognani F, Muller A, Dubost V, Luisier R, Del Rio Espinola A, Vitry V, Unterberger EB, Thomson JP, (2013) Identification of Dlk1-Dio3 imprinted gene cluster noncoding RNAs as novel candidate biomarkers for liver tumor promotion. Toxicol Sci 131: 375–386. 10.1093/toxsci/kfs303 23091169

[bib49] Lempiainen H, Muller A, Brasa S, Teo SS, Roloff TC, Morawiec L, Zamurovic N, Vicart A, Funhoff E, Couttet P, (2011) Phenobarbital mediates an epigenetic switch at the constitutive androstane receptor (CAR) target gene Cyp2b10 in the liver of B6C3F1 mice. PLoS One 6: e18216 10.1371/journal.pone.0018216 21455306 PMC3063791

[bib50] Lewis L, Crawford GE, Furey TS, Rusyn I (2017) Genetic and epigenetic determinants of inter-individual variability in responses to toxicants. Curr Opin Toxicol 6: 50–59. 10.1016/j.cotox.2017.08.006 29276797 PMC5739339

[bib51] Li H, Handsaker B, Wysoker A, Fennell T, Ruan J, Homer N, Marth G, Abecasis G, Durbin R, Genome Project Data Processing Subgroup, (2009) The sequence alignment/map format and SAMtools. Bioinformatics 25: 2078–2079. 10.1093/bioinformatics/btp352 19505943 PMC2723002

[bib52] Li Z, Tuteja G, Schug J, Kaestner KH (2012) Foxa1 and Foxa2 are essential for sexual dimorphism in liver cancer. Cell 148: 72–83. 10.1016/j.cell.2011.11.026 22265403 PMC3266536

[bib53] Ling G, Waxman DJ (2013a) DNase I digestion of isolated nulcei for genome-wide mapping of DNase hypersensitivity sites in chromatin. Methods Mol Biol 977: 21–33. 10.1007/978-1-62703-284-1_3 23436351 PMC3889470

[bib54] Ling G, Waxman DJ (2013b) Isolation of nuclei for use in genome-wide DNase hypersensitivity assays to probe chromatin structure. Methods Mol Biol 977: 13–19. 10.1007/978-1-62703-284-1_2 23436350 PMC3815455

[bib55] Liu H, Fergusson MM, Wu JJ, Rovira II, Liu J, Gavrilova O, Lu T, Bao J, Han D, Sack MN, (2011) Wnt signaling regulates hepatic metabolism. Sci Signal 4: ra6 10.1126/scisignal.2002099 21285411 PMC3147298

[bib56] Liu L, Luo GZ, Yang W, Zhao X, Zheng Q, Lv Z, Li W, Wu HJ, Wang L, Wang XJ, (2010) Activation of the imprinted Dlk1-Dio3 region correlates with pluripotency levels of mouse stem cells. J Biol Chem 285: 19483–19490. 10.1074/jbc.m110.131995 20382743 PMC2885227

[bib57] Luisier R, Lempiainen H, Scherbichler N, Braeuning A, Geissler M, Dubost V, Muller A, Scheer N, Chibout SD, Hara H, (2014a) Phenobarbital induces cell cycle transcriptional responses in mouse liver humanized for constitutive androstane and pregnane x receptors. Toxicol Sci 139: 501–511. 10.1093/toxsci/kfu038 24690595

[bib58] Luisier R, Unterberger EB, Goodman JI, Schwarz M, Moggs J, Terranova R, van Nimwegen E (2014b) Computational modeling identifies key gene regulatory interactions underlying phenobarbital-mediated tumor promotion. Nucleic Acids Res 42: 4180–4195. 10.1093/nar/gkt1415 24464994 PMC3985636

[bib59] Luk JM, Burchard J, Zhang C, Liu AM, Wong KF, Shek FH, Lee NP, Fan ST, Poon RT, Ivanovska I, (2011) DLK1-DIO3 genomic imprinted microRNA cluster at 14q32.2 defines a stemlike subtype of hepatocellular carcinoma associated with poor survival. J Biol Chem 286: 30706–30713. 10.1074/jbc.m111.229831 21737452 PMC3162431

[bib60] Luo Q, Wang CQ, Yang LY, Gao XM, Sun HT, Zhang Y, Zhang KL, Zhu Y, Zheng Y, Sheng YY, (2018) FOXQ1/NDRG1 axis exacerbates hepatocellular carcinoma initiation via enhancing crosstalk between fibroblasts and tumor cells. Cancer Lett 417: 21–34. 10.1016/j.canlet.2017.12.021 29248714

[bib61] Madapura HS, Nagy N, Ujvari D, Kallas T, Krohnke MCL, Amu S, Bjorkholm M, Stenke L, Mandal PK, McMurray JS, (2017) Interferon gamma is a STAT1-dependent direct inducer of BCL6 expression in imatinib-treated chronic myeloid leukemia cells. Oncogene 36: 4619–4628. 10.1038/onc.2017.85 28368400

[bib62] Maurano MT, Humbert R, Rynes E, Thurman RE, Haugen E, Wang H, Reynolds AP, Sandstrom R, Qu H, Brody J, (2012) Systematic localization of common disease-associated variation in regulatory DNA. Science 337: 1190–1195. 10.1126/science.1222794 22955828 PMC3771521

[bib63] McCarthy DJ, Chen Y, Smyth GK (2012) Differential expression analysis of multifactor RNA-Seq experiments with respect to biological variation. Nucleic Acids Res 40: 4288–4297. 10.1093/nar/gks042 22287627 PMC3378882

[bib64] McConnell BB, Yang VW (2010) Mammalian Kruppel-like factors in health and diseases. Physiol Rev 90: 1337–1381. 10.1152/physrev.00058.2009 20959618 PMC2975554

[bib65] McKenna A, Hanna M, Banks E, Sivachenko A, Cibulskis K, Kernytsky A, Garimella K, Altshuler D, Gabriel S, Daly M, (2010) The genome analysis toolkit: A MapReduce framework for analyzing next-generation DNA sequencing data. Genome Res 20: 1297–1303. 10.1101/gr.107524.110 20644199 PMC2928508

[bib66] Mifsud B, Tavares-Cadete F, Young AN, Sugar R, Schoenfelder S, Ferreira L, Wingett SW, Andrews S, Grey W, Ewels PA, (2015) Mapping long-range promoter contacts in human cells with high-resolution capture Hi-C. Nat Genet 47: 598–606. 10.1038/ng.3286 25938943

[bib67] Nguyen NTT, Contreras-Moreira B, Castro-Mondragon JA, Santana-Garcia W, Ossio R, Robles-Espinoza CD, Bahin M, Collombet S, Vincens P, Thieffry D, (2018) RSAT 2018: Regulatory sequence analysis tools 20th anniversary. Nucleic Acids Res 46: W209–W214. 10.1093/nar/gkx1003 29722874 PMC6030903

[bib68] Nowak K, Stein G, Powell E, He LM, Naik S, Morris J, Marlow S, Davis TL (2011) Establishment of paternal allele-specific DNA methylation at the imprinted mouse Gtl2 locus. Epigenetics 6: 1012–1020. 10.4161/epi.6.8.16075 21725202 PMC3359488

[bib69] Oshida K, Waxman DJ, Corton JC (2016) Chemical and hormonal effects on STAT5b-dependent sexual dimorphism of the liver Transcriptome. PLoS One 11: e0150284 10.1371/journal.pone.0150284 26959237 PMC4784907

[bib70] Pennacchio LA, Bickmore W, Dean A, Nobrega MA, Bejerano G (2013) Enhancers: Five essential questions. Nat Rev Genet 14: 288–295. 10.1038/nrg3458 23503198 PMC4445073

[bib71] Peraino C, Fry RJ, Staffeldt E (1973) Brief communication: Enhancement of spontaneous hepatic tumorigenesis in C3H mice by dietary phenobarbital. J Natl Cancer Inst 51: 1349–1350. 10.1093/jnci/51.4.1349 4745865

[bib72] Phillips JM, Burgoon LD, Goodman JI (2009a) The constitutive active/androstane receptor facilitates unique phenobarbital-induced expression changes of genes involved in key pathways in precancerous liver and liver tumors. Toxicol Sci 110: 319–333. 10.1093/toxsci/kfp108 19482888 PMC2708600

[bib73] Phillips JM, Burgoon LD, Goodman JI (2009b) Phenobarbital elicits unique, early changes in the expression of hepatic genes that affect critical pathways in tumor-prone B6C3F1 mice. Toxicol Sci 109: 193–205. 10.1093/toxsci/kfp050 19270015 PMC2683922

[bib74] Pogribny IP, Rusyn I (2014) Role of epigenetic aberrations in the development and progression of human hepatocellular carcinoma. Cancer Lett 342: 223–230. 10.1016/j.canlet.2012.01.038 22306342 PMC3971756

[bib75] Pouche L, Vitobello A, Romer M, Glogovac M, MacLeod AK, Ellinger-Ziegelbauer H, Westphal M, Dubost V, Stiehl DP, Dumotier B, (2017) Xenobiotic CAR activators induce Dlk1-Dio3 locus non-coding RNA expression in mouse liver. Toxicol Sci 158: 367–378. 10.1093/toxsci/kfx104 28541575

[bib76] Quinlan AR (2014) BEDTools: The Swiss-Army tool for genome feature analysis. Curr Protoc Bioinformatics 47: 1–34. 10.1002/0471250953.bi1112s47 25199790 PMC4213956

[bib77] Ram O, Goren A, Amit I, Shoresh N, Yosef N, Ernst J, Kellis M, Gymrek M, Issner R, Coyne M, (2011) Combinatorial patterning of chromatin regulators uncovered by genome-wide location analysis in human cells. Cell 147: 1628–1639. 10.1016/j.cell.2011.09.057 22196736 PMC3312319

[bib78] Rignall B, Braeuning A, Buchmann A, Schwarz M (2011) Tumor formation in liver of conditional beta-catenin-deficient mice exposed to a diethylnitrosamine/phenobarbital tumor promotion regimen. Carcinogenesis 32: 52–57. 10.1093/carcin/bgq226 21047994

[bib79] Robinson JT, Thorvaldsdottir H, Winckler W, Guttman M, Lander ES, Getz G, Mesirov JP (2011) Integrative genomics viewer. Nat Biotechnol 29: 24–26. 10.1038/nbt.1754 21221095 PMC3346182

[bib80] Robinson MD, McCarthy DJ, Smyth GK (2010) edgeR: A Bioconductor package for differential expression analysis of digital gene expression data. Bioinformatics 26: 139–140. 10.1093/bioinformatics/btp616 19910308 PMC2796818

[bib81] Ross-Innes CS, Stark R, Teschendorff AE, Holmes KA, Ali HR, Dunning MJ, Brown GD, Gojis O, Ellis IO, Green AR, (2012) Differential oestrogen receptor binding is associated with clinical outcome in breast cancer. Nature 481: 389–393. 10.1038/nature10730 22217937 PMC3272464

[bib82] Santos NP, Colaco AA, Oliveira PA (2017) Animal models as a tool in hepatocellular carcinoma research: A review. Tumour Biol 39: 1010428317695923 10.1177/1010428317695923 28347231

[bib83] Shen Y, Yue F, McCleary DF, Ye Z, Edsall L, Kuan S, Wagner U, Dixon J, Lee L, Lobanenkov VV, (2012) A map of the cis-regulatory sequences in the mouse genome. Nature 488: 116–120. 10.1038/nature11243 22763441 PMC4041622

[bib84] Shlyueva D, Stampfel G, Stark A (2014) Transcriptional enhancers: From properties to genome-wide predictions. Nat Rev Genet 15: 272–286. 10.1038/nrg3682 24614317

[bib85] Soldner F, Stelzer Y, Shivalila CS, Abraham BJ, Latourelle JC, Barrasa MI, Goldmann J, Myers RH, Young RA, Jaenisch R (2016) Parkinson-associated risk variant in distal enhancer of alpha-synuclein modulates target gene expression. Nature 533: 95–99. 10.1038/nature17939 27096366 PMC5042324

[bib86] Stadtfeld M, Apostolou E, Akutsu H, Fukuda A, Follett P, Natesan S, Kono T, Shioda T, Hochedlinger K (2010) Aberrant silencing of imprinted genes on chromosome 12qF1 in mouse induced pluripotent stem cells. Nature 465: 175–181. 10.1038/nature09017 20418860 PMC3987905

[bib87] Sur I, Taipale J (2016) The role of enhancers in cancer. Nat Rev Cancer 16: 483–493. 10.1038/nrc.2016.62 27364481

[bib88] Szyf M (2007) The dynamic epigenome and its implications in toxicology. Toxicol Sci 100: 7–23. 10.1093/toxsci/kfm177 17675334

[bib89] Takahashi K, Tanabe K, Ohnuki M, Narita M, Ichisaka T, Tomoda K, Yamanaka S (2007) Induction of pluripotent stem cells from adult human fibroblasts by defined factors. Cell 131: 861–872. 10.1016/j.cell.2007.11.019 18035408

[bib90] Thomson JP, Hunter JM, Lempiainen H, Muller A, Terranova R, Moggs JG, Meehan RR (2013) Dynamic changes in 5-hydroxymethylation signatures underpin early and late events in drug exposed liver. Nucleic Acids Res 41: 5639–5654. 10.1093/nar/gkt232 23598998 PMC3675467

[bib91] Thomson JP, Lempiainen H, Hackett JA, Nestor CE, Muller A, Bolognani F, Oakeley EJ, Schubeler D, Terranova R, Reinhardt D, (2012) Non-genotoxic carcinogen exposure induces defined changes in the 5-hydroxymethylome. Genome Biol 13: R93 10.1186/gb-2012-13-10-r93 23034186 PMC3491421

[bib92] Thomson JP, Moggs JG, Wolf CR, Meehan RR (2014) Epigenetic profiles as defined signatures of xenobiotic exposure. Mutat Res Genet Toxicol Environ Mutagen 764-765: 3–9. 10.1016/j.mrgentox.2013.08.007 24001620

[bib93] Thomson JP, Ottaviano R, Unterberger EB, Lempiainen H, Muller A, Terranova R, Illingworth RS, Webb S, Kerr AR, Lyall MJ, (2016) Loss of Tet1-associated 5-hydroxymethylcytosine is concomitant with aberrant promoter hypermethylation in liver cancer. Cancer Res 76: 3097–3108. 10.1158/0008-5472.can-15-1910 27197233 PMC5021200

[bib94] Thorvaldsdottir H, Robinson JT, Mesirov JP (2013) Integrative genomics viewer (IGV): High-performance genomics data visualization and exploration. Brief Bioinform 14: 178–192. 10.1093/bib/bbs017 22517427 PMC3603213

[bib95] Thurman RE, Rynes E, Humbert R, Vierstra J, Maurano MT, Haugen E, Sheffield NC, Stergachis AB, Wang H, Vernot B, (2012) The accessible chromatin landscape of the human genome. Nature 489: 75–82. 10.1038/nature11232 22955617 PMC3721348

[bib96] Vicente-Duenas C, Hauer J, Cobaleda C, Borkhardt A, Sanchez-Garcia I (2018) Epigenetic priming in cancer initiation. Trends Cancer 4: 408–417. 10.1016/j.trecan.2018.04.007 29860985

[bib97] Vierstra J, Wang H, John S, Sandstrom R, Stamatoyannopoulos JA (2014) Coupling transcription factor occupancy to nucleosome architecture with DNase-FLASH. Nat Methods 11: 66–72. 10.1038/nmeth.2713 24185839

[bib98] Wang B, Zhao L, Fish M, Logan CY, Nusse R (2015a) Self-renewing diploid Axin2(+) cells fuel homeostatic renewal of the liver. Nature 524: 180–185. 10.1038/nature14863 26245375 PMC4589224

[bib99] Wang H, Lafdil F, Wang L, Park O, Yin S, Niu J, Miller AM, Sun Z, Gao B (2011) Hepatoprotective versus oncogenic functions of STAT3 in liver tumorigenesis. Am J Pathol 179: 714–724. 10.1016/j.ajpath.2011.05.005 21684247 PMC3157203

[bib100] Wang J, Dong L, Xu L, Chu ES, Chen Y, Shen J, Li X, Wong CC, Sung JJ, Yu J (2014) B cell CLL/lymphoma 6 member B inhibits hepatocellular carcinoma metastases in vitro and in mice. Cancer Lett 355: 192–200. 10.1016/j.canlet.2014.08.025 25218345

[bib101] Wang W, Huang P, Wu P, Kong R, Xu J, Zhang L, Yang Q, Xie Q, Zhang L, Zhou X, (2015b) BCL6B expression in hepatocellular carcinoma and its efficacy in the inhibition of liver damage and fibrogenesis. Oncotarget 6: 20252–20265. 10.18632/oncotarget.3857 25970780 PMC4653002

[bib102] Welch RP, Lee C, Imbriano PM, Patil S, Weymouth TE, Smith RA, Scott LJ, Sartor MA (2014) ChIP-enrich: Gene set enrichment testing for ChIP-seq data. Nucleic Acids Res 42: e105 10.1093/nar/gku463 24878920 PMC4117744

[bib103] Xu S, Shu P, Zou S, Shen X, Qu Y, Zhang Y, Sun K, Zhang J (2018) NFATc1 is a tumor suppressor in hepatocellular carcinoma and induces tumor cell apoptosis by activating the FasL-mediated extrinsic signaling pathway. Cancer Med 7: 4701–4717. 10.1002/cam4.1716 30085405 PMC6143940

[bib104] Yoshida K, Murata M, Yamaguchi T, Matsuzaki K (2014) TGF-beta/Smad signaling during hepatic fibro-carcinogenesis (review). Int J Oncol 45: 1363–1371. 10.3892/ijo.2014.2552 25050845 PMC4151811

[bib105] Zhang Y, Liu T, Meyer CA, Eeckhoute J, Johnson DS, Bernstein BE, Nusbaum C, Myers RM, Brown M, Li W, (2008) Model-based analysis of chip-seq (MACS). Genome Biol 9: R137 10.1186/gb-2008-9-9-r137 18798982 PMC2592715

[bib106] Zhang Y, Wong CH, Birnbaum RY, Li G, Favaro R, Ngan CY, Lim J, Tai E, Poh HM, Wong E, (2013) Chromatin connectivity maps reveal dynamic promoter-enhancer long-range associations. Nature 504: 306–310. 10.1038/nature12716 24213634 PMC3954713

[bib107] Zhu LJ (2013) Integrative analysis of ChIP-chip and ChIP-seq dataset. Methods Mol Biol 1067: 105–124. 10.1007/978-1-62703-607-8_8 23975789

[bib108] Zhu LJ, Gazin C, Lawson ND, Pages H, Lin SM, Lapointe DS, Green MR (2010) ChIPpeakAnno: A bioconductor package to annotate ChIP-seq and ChIP-chip data. BMC Bioinformatics 11: 237 10.1186/1471-2105-11-237 20459804 PMC3098059

