# Supplementary material for: Drug-induced chromatin accessibility changes associate with sensitivity to liver tumor promotion
Source: Life Sci Alliance. 2019 Oct 15;2(5):e201900461. doi: 10.26508/lsa.201900461 (PMC6795216; doi:10.26508/lsa.201900461)
Supplement: Supplementary file 1 [file LSA-2019-00461_TableS1.docx]

**Table S1 – Cross-strain comparison of Phenobarbital-mediated liver tumor promotion incidence in mice**

| **Strain** | **Duration** | **PB Dose/Route** | **Incidence of Neoplasia** | | | |
| --- | --- | --- | --- | --- | --- | --- |
|  |  |  | **Control** | **PB** | **DEN Initiated** | **Initiated + PB** |
| **Adapted from: Becker FF,1982 [1]** | | | | | | |
| C57BL/6N | 72 weeks | 500 ppm in drinking water | (0/16) 0% | (0/16) 0% | n.d | n.d |
| C3H/HeN | 52 weeks |  | (10/16) 61% | (16/16) 100% | n.d | n.d |
| B6C3F1 | 52 weeks |  | (5/16) 29% | (16/16) 100% | n.d | n.d |
| **Adapted from: Goldsworthy TL & Fransson-Steen R, 2002** **[2]** | | | | | | |
| C57BL/6N | 36 weeks | 500 ppm in drinking water | (0/15) 0% | (0/13) 0% | (5/14) 36% | (6/13) 46% |
| C3H/HeN | 36 weeks |  | (4/13) 31% | (7/10) 70% | (13/13) 100% | (9/9) 100% |
| B6C3F1 | 36 weeks |  | (2/15) 13% | (2/15) 13% | (13/15) 87% | (12/14) 86% |
| **Adapted from: Bursch W et al., 2005 [3]** | | | | | | |
| C57BL/6J | 40 weeks | 500-700 ppm in diet | n.d | n.d | (1/7) 14% | (1/5) 20% |
|  | 52 weeks |  | (1/7) 17% | (0/6) 0% | (6/8) 75% | (5/8) 63% |
|  | 76 weeks |  | (1/4) 25% | (0/4) 0% | (9/12) 75% | (10/12) 83% |
| C3H/He | 40 weeks |  | n.d | n.d | (5/6) 83% | (7/7) 100% |
|  | 52 weeks |  | (3/6) 50% | (1/6) 17% | (8/8) 100% | (11/11) 100% |
|  | 76 weeks |  | (3/5) 60% | (6/6) 100% | (7/7) 100% | n.a |
| B6C3F1 | 40 weeks |  | n.d | n.d | (2/8) 25% | (3/8) 38% |
|  | 52 weeks |  | (0/8) 0% | (3/6) 50% | (3/10) 30% | (10/10)100% |
|  | 76 weeks |  | (0/5) 0% | (3/7) 43% | (13/15) 87% | (9/9) 100% |

**References**

1. Becker FF: **Morphological classification of mouse liver tumors based on biological characteristics.** *Cancer Res* 1982, **42:**3918-3923.

2. Goldsworthy TL, Fransson-Steen R: **Quantitation of the cancer process in C57BL/6J, B6C3F1 and C3H/HeJ mice.** *Toxicol Pathol* 2002, **30:**97-105.

3. Bursch W, Chabicovsky M, Wastl U, Grasl-Kraupp B, Bukowska K, Taper H, Schulte-Hermann R: **Apoptosis in stages of mouse hepatocarcinogenesis: failure to counterbalance cell proliferation and to account for strain differences in tumor susceptibility.** *Toxicol Sci* 2005, **85:**515-529.
